# Reassessing the adverse event profiles of levetiracetam and brivaracetam: a systematic review and meta-analysis

**DOI:** 10.1007/s00415-026-13746-9

**Published:** 2026-03-13

**Authors:** Emma Fröling, Mariel Morales Sahm, Marcel Schmude, Charlotte P. Assies, Michael Wittenberg, Felix Rosenow, Felix Bermpohl, Thomas G. Riemer

**Affiliations:** 1 Department of Psychiatry and Psychotherapy, Berlin Institute of Health, Charité – Universitätsmedizin Berlin, Corporate Member of Freie Universität Berlin, Humboldt-Universität zu Berlin, 10115 Berlin, Germany; 2 Institute of Clinical Pharmacology and Toxicology, Berlin Institute of Health, Charité – Universitätsmedizin Berlin, Corporate Member of Freie Universität Berlin, Humboldt-Universität zu Berlin, 10117 Berlin, Germany; 3 Institute of Psychology, Heidelberg University, 69117 Heidelberg, Germany; 4 Department Marburg, Faculty of Medicine, Coordinating Center of Clinical Trials, Marburg University, 35043 Marburg, Germany; 5 Department of Neurology, Epilepsy Center Frankfurt Rhine-Main, Universitätsmedizin Frankfurt, Goethe-Universität Frankfurt, 60590 Frankfurt, Germany

**Keywords:** Antiseizure medication, Epilepsy, Anticonvulsants, Safety profile

## Abstract

**Objective:**

To synthesize adverse event (AE) data from randomized controlled trials (RCTs) of levetiracetam (LEV) and brivaracetam (BRV), compare their safety profiles, and identify moderators of AE risk.

**Methods:**

We conducted a systematic review and meta-analysis of RCTs of LEV or BRV, searching PubMed, Web of Science, and ClinicalTrials.gov to August 2025. AE frequencies were summarized qualitatively, and random-effects meta-analyses compared LEV and BRV with placebo. Additional analyses compared LEV and BRV and assessed moderators. PROSPERO: CRD42023491050, CRD420251003207.

**Results:**

Ninety-six RCTs including 7145 patients exposed to LEV and 2549 to BRV were analyzed. Qualitative synthesis identified headache, somnolence, dizziness, and fatigue as the most common AEs, with higher frequencies of some psychiatric AEs (e.g., irritability, aggression) reported for LEV. Meta-analyses showed increased somnolence with both LEV (OR 1.80, 95% CI [1.41–2.30]) and BRV (OR 1.86, 95% CI [1.33–2.61]). LEV was additionally associated with irritability (OR 2.55, 95% CI [1.41–4.63]) and asthenia (OR 1.71, 95% CI [1.15–2.54]), whereas BRV was associated with dizziness (OR 1.75, 95% CI [1.24–2.46]) and fatigue (OR 2.14, 95% CI [1.43–3.20]). Few moderators of AE risk were identified, and no significant differences emerged between LEV and BRV in indirect comparisons.

**Conclusion:**

LEV and BRV show favorable safety profiles that appear comparable based on meta-analytic findings, despite higher rates of some psychiatric AEs with LEV on a descriptive level. Both remain safe treatment options, though larger head-to-head trials are needed to provide definitive comparative evidence.

**Supplementary Information:**

The online version contains supplementary material available at 10.1007/s00415-026-13746-9.

## Introduction

Epilepsy affects approximately 65 million individuals worldwide, making it one of the most prevalent neurological disorders [1]. As such, it imposes a substantial global burden, particularly in terms of years lived with disability [2]. While antiseizure medications (ASMs) enable seizure control in up to 70% of patients [3], treatment tolerability remains a significant concern, as adverse events (AEs) can compromise adherence and quality of life [3, 4]. AEs not only diminish patients' psychosocial well-being but may also lead to emergency consultations or treatment discontinuation [5, 6], ultimately compromising long-term seizure control. Among the widely used newer ASMs, levetiracetam (LEV) and brivaracetam (BRV) exemplify the tension between efficacy and tolerability. Given that many patients require lifelong therapy, tolerability issues are of major clinical relevance.

LEV, a second-generation ASM, exerts its antiepileptic effects via binding to synaptic vesicle protein 2 (SV2A). It plays a pivotal role in the treatment of epilepsy, particularly as a first-line option for focal seizures, owing to its efficacy, minimal drug interactions, and favorable pharmacokinetics [7, 8]. However, LEV is associated with a distinct AE profile, which may lead to decreased adherence or discontinuation [9]. Psychiatric and behavioral AEs, such as irritability and mood disturbances, are particularly well recognized [10]. A meta-analysis on the AE profile also linked neurological and systemic AEs such as somnolence, dizziness, asthenia/fatigue, and nasopharyngitis to LEV use. Notably, these events may occur independently of dose [11].

BRV is a propyl analog of LEV with a 15–30-fold higher binding affinity for SV2A [12]. Across randomized controlled trials (RCTs), BRV demonstrated efficacy comparable to LEV in focal seizures, with consistent efficacy observed in special populations such as generalized epilepsy, post-stroke epilepsy, and elderly patients [13]. Since its approval in 2016, BRV has been included in international guidelines and is increasingly used as a second-line strategy for mono- or add-on therapy [14]. Regarding AEs, a meta-analysis reported an increased risk of dizziness and fatigue, but a reduced risk of back pain compared to placebo [15]. Overall, its safety profile resembles that of LEV, although BRV appears to carry a lower risk of psychiatric and behavioral AEs, yet a higher risk of dizziness [13, 16, 17].

While both LEV and BRV act via SV2A, important pharmacological distinctions may account for differences in tolerability. In addition to its higher binding affinity for SV2A, BRV exhibits greater lipophilicity, resulting in faster brain penetration and more rapid SV2A binding [18]. Evidence also suggests that LEV and BRV engage distinct SV2A binding sites [19]. Beyond SV2A, LEV has been shown to affect high-voltage-activated calcium currents and neurotransmitter balance, whereas BRV displays additional inhibitory activity at neuronal voltage-dependent sodium channels [20]. Such differences provide a mechanistic rationale for potential divergences in their AE profiles.

AEs of both LEV and BRV have already been examined in several systematic reviews and meta-analyses. Many recent studies focus on specific AE categories (e.g., psychiatric and behavioral disturbances [21]) or selected patient populations such as children [22]. While such targeted analyses provide valuable insights, they do not capture the full spectrum of tolerability across diverse clinical contexts. Broader assessments of the overall AE profile, on the other hand, are nearly a decade old and therefore do not account for more recent RCTs [11, 15]. As a result, the current evidence base remains fragmented, underscoring the need for a comprehensive synthesis to better inform patient-centered treatment decisions.

This systematic review and meta-analysis therefore aims to (1) characterize the full range and frequencies of AEs reported with LEV and BRV in RCTs, (2) determine whether individual AEs or overall safety profiles differ in clinically relevant ways between the two drugs, and (3) identify treatment- and population-related moderators of AE risk for each agent.

## Methods

### Protocol and registration

This systematic review and meta-analysis was conducted in accordance with the updated guideline for reporting systematic reviews of PRISMA 2020 [23]. The study was pre-registered in PROSPERO: CRD42023491050 and CRD420251003207.

### Search strategy and eligibility criteria (information sources)

A systematic search was conducted in PubMed, Web of Science, Clinicaltrials.gov, and the Vivli platform for global clinical research data from database inception to 16th August 2025. Two independent literature searches were conducted using the keywords “Levetiracetam” and “Brivaracetam”. Only studies published in German and English were considered. In case of incomplete or missing data, corresponding authors or principal investigators were contacted. Additionally, European Medicine Agency and Food and Drug Administration drug approval packages were screened and eligible studies included in the analysis. Comprehensive inclusion and exclusion criteria can be found in Table S1. Briefly, prospective RCTs in humans receiving LEV or BRV monotherapy in at least one study arm, with a minimum treatment duration of 14 consecutive days, were included. Notably, we included RCTs across all clinical indications to maximize statistical power and provide a comprehensive characterization of the AE profiles for LEV and BRV. All inclusion criteria were defined a priori.

### Study selection, data extraction, and data synthesis

Two reviewers (E.F. and M.M.S.) independently assessed study eligibility and extracted data on study design, treatment (type, duration, dose, titration schemes), patient characteristics (sample size, age, indication, background medication), and AEs (symptom, frequency, assessment methodology). Terminological inconsistencies in the reported AEs across studies were noted. Therefore, AEs were categorized according to Medical Dictionary for Regulatory Activities (MedDRA) guidelines by a multidisciplinary team consisting of pharmacists and clinical pharmacologists (E.F., M.M.S., T.G.R.). Symptoms were clustered if (1) they were synonymous or are listed under the same preferred name according to MedDRA Version 27.0, and (2) did not co-occur in the same study [24].

### Quality assessment

Two reviewers (E.F. and M.M.S.) performed a quality assessment of the included studies by using the revised Cochrane risk of bias assessment tool [25]. The quality of AE collection and reporting was evaluated using a previously validated checklist [26]. Disagreements were resolved through discussion with a third reviewer (T.G.R.).

### Evaluation of AE profiles of LEV and BRV

#### Qualitative analysis

A qualitative analysis was conducted to identify the most frequently reported AEs associated with LEV and BRV therapy, respectively. All studies regardless of the comparator were included. AEs reported in more than five studies were included in this analysis. The events were stratified by target dose, and results for placebo were reported for comparison. For each study, the proportion of participants experiencing a given AE was calculated (affected patients/patients at risk) and then aggregated by event and dose group to determine the weighted mean frequency.

#### Quantitative analysis

The main objective of the meta-analyses was to compare the occurrence of AEs between LEV or BRV against placebo. Meta-analyses were performed using Cochrane RevMan (Review Manager) Version 9.5.2 if at least five parallel-group, placebo-controlled trials reported the respective AE to ensure statistical power [27, 28]. All daily dosage regimes of LEV or BRV were aggregated, respectively. Odds ratios with 95% confidence intervals were calculated by using random-effects models considering heterogeneity in the study population and design. Statistical significance was defined as *p* ≤ 0.05. Odds ratios were employed to quantify the strength of association between drug exposure and the occurrence of AEs, since ORs allow direct comparisons of the events making them particularly relevant while analysing low event rates and considering different studies with varying baseline risks [29]. Heterogeneity was assessed using the I^2^ statistic following Cochrane Handbook threshold recommendations [30]. Publication bias was evaluated through funnel plot inspection for comparisons supported by  at least tenstudies. Unpublished data were explicitly flagged to detect potential distortions.

#### Sensitivity analyses

Seven sensitivity analyses were conducted to examine the robustness of the findings: Exclusion of the study with the greatest weight; exclusion of studies with a duration of fewer than 8 weeks; exclusion of studies with a high risk of bias based on Cochrane risk of bias tool; selective inclusion of studies assessing frequencies of AEs in an active or passive manner, respectively; exclusion of studies with any indication of industry sponsorship and exclusion of pooled data; and exclusion of unpublished data sets. Rationales are presented in Table S2.

### Comparison of LEV and BRV

AE frequencies for LEV and BRV were first compared descriptively, using aggregated relative frequencies of all AEs that were reported in a minimum of five studies. Safety profiles of LEV and BRV were then compared both indirectly and directly. Indirect comparisons were based on their respective meta-analyses, with subgroup analyses against placebo conducted after pooling the datasets if for both components an analysis was available. In addition, evidence from a recently published head-to-head trial directly comparing LEV and BRV was incorporated to complement and validate the indirect findings.

### Assessment of moderators of AE risks

#### Analysis of dose dependencies

To explore potential dose-AE relationships, a threefold analytic strategy was applied:

- **Qualitative dose–frequency assessment**: To explore overall patterns of dose dependency, AE frequencies were plotted against the reported target doses of LEV and BRV. For this assessment, the clinically relevant dose ranges were considered (LEV: 1000–3000 mg; BRV: 50–200 mg). Descriptive comparisons were performed only when data from at least three studies per dose arm were available for the respective AE, to ensure more reliable and stable estimates.
- **Dose–response quantification via meta-regression**: To formally assess the association between dose and AE risk, meta-regressions were conducted in R Statistical Software (v4.5.0) using the metafor package, version 4.8–0 [31, 32]. This approach was restricted to studies included in the meta-analyses that reported a clearly defined target dose, thereby providing a statistically rigorous estimate of dose–AE relationships.
- **High- versus low-dose comparisons**: Additional meta-analyses were performed comparing the highest versus the lowest target dose arms of trials that reported AEs separately for at least two dose levels. This ensured within-study comparability and mitigated confounding across different trial designs.

#### Subgroup analyses

Subgroups analyses were conducted to evaluate the influence of further moderators on the occurrence of AEs. Moderators of interest were patients age, indication of therapy, dose titration schemes, and the presence of background medication. Subgroup-analyses were performed if at least two studies per group were available.

## Results

### Search results

**Fig. 1:**
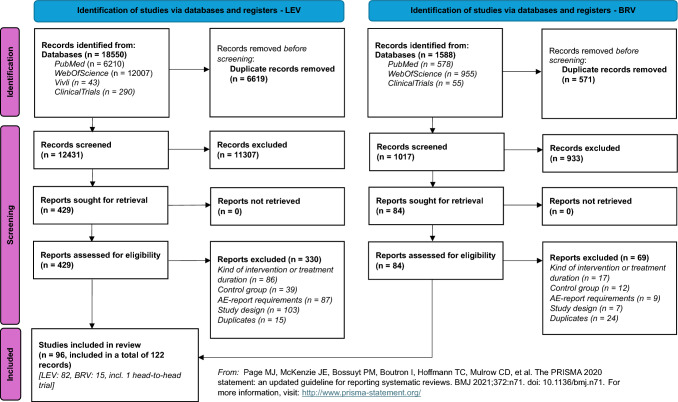
Study selection process

After removal of duplicates, a total of 13,448 records were identified. Following title and abstract screening, 12,240 records were excluded. The remaining 513 full-text articles were assessed for eligibility. Ultimately, 96 studies (LEV: 82; BRV: 15, including 1 head-to-head-trial) were included in the descriptive qualitative analysis, of which 48 were parallel, placebo-controlled (LEV: 35; BRV: 13). Additional data were obtained from study authors in 10 instances. Details on the study selection process can be found in Fig. 1.

### Studies

A total of 96 studies were included in the qualitative analysis (LEV: 82; BRV: 15; includes 1 LEV–BRV head-to-head trial), encompassing a total of 7145 patients exposed to LEV and 2549 to BRV. Among these, 59 trials were placebo-controlled (LEV: 46; BRV: 13), 81 had a parallel-group design (LEV: 68; BRV: 15), and 15 studies with a cross-over design were identified only with LEV. Studies using active comparators were found with both drugs (LEV: 39; BRV: 4), including records comparing different target doses of BRV and LEV or with other ASM. More details are shown in Tables S3 and S4. A total of 21 studies were open-label (LEV: 21; BRV: 0). The majority of trials investigated patients with epilepsy/seizure prophylaxis (LEV: 55; BRV: 13). Other common indications were various kinds of pain (LEV: 6; BRV: 2), tremor (LEV: 5; BRV: 0) and cognitive impairment (LEV: 5; BRV: 0). A high risk of bias was identified in 30 studies (LEV: 29; BRV: 1), and AE reporting quality was limited, with only 14 records providing AE frequencies without applying a reporting threshold (LEV: 10; BRV: 4). An AE assessment method was described in 62 studies of which 13 studies used an active one. The overview of results of Risk of Bias rating is shown in Figs. S1, S2. 12 trials were unpublished (LEV: 10; BRV: 2), and an industry sponsorship was indicated in 43 records (LEV: 29; BRV: 14).

### Evaluation of AE profiles of LEV and BRV

#### Qualitative analysis

The ten most frequently reported events were: (1) LEV: headache (794 cases out of 5729 exposed patients reported in 52 trials), somnolence (733 out of 5768 in 47 trials), dizziness (554 out of 5754 in 57 trials), nasopharyngitis (406 out of 3631 in 22 trials), fatigue (401 out of 3268 in 29 trials), nausea (238 out of 4645 in 38 trials), diarrhea (178 out of 4020 in 26 trials), irritability (156 out of 2498 in 31 trials), depression (155 out of 3300 in 19 trials) and asthenia (137 out of 2151 in 16 trials). (2) BRV: somnolence (349 cases out of 2426 exposed patients reported in 12 trials), headache (249 out of 2549 in 15 trials), dizziness (231 out of 2351 in 13 trials), fatigue (165 out of 2052 in 12 trials), nausea (93 out of 2035 in 11 trials), nasopharyngitis (91 out of 2084 in 10 trials), convulsion (64 out of 1939 in 10 trials), urinary tract infection (54 out of 1725 in 9 trials), irritability (45 out of 1790 in 8 trials) and depression (42 out of 1905 in 9 trials). These results are presented in Fig. 4. Frequencies for AEs reported in at least five studies, stratified by target dose and comparator, are provided in Tables S5, S6. All reported AEs can be found in Tables S7 and S8.

#### Quantitative analysis

Meta-analyses were conducted for 22 AEs for LEV based on data from 35 trials and 11 AEs for BRV based on 11 trials. Statistically significant pooled effects versus placebo were observed for the following AEs, indicating a different risk under anticonvulsive therapy: For LEV somnolence (OR 1.80, 95% CI [1.41, 2.30], *p*-value < 0.00001, I_2_ = 0%), irritability (OR 2.55, 95% CI [1.41, 4.63], *p*-value = 0.002, I_2_ = 0%) and asthenia (OR 1.71, 95% CI [1.15, 2.54], *p*-value = 0.008, I_2_ = 0%), and for BRV somnolence (OR 1.86, 95% CI [1.33, 2.61, *p*-value = 0.0003, I_2_ = 18%), dizziness (OR 1.75, 95% CI [1.24, 2.46], *p*-value = 0.001, I_2_ = 9%) and fatigue (OR 2.14, 95% CI [1.43, 3.20], *p*-value = 0.0002, I_2_ = 0%).

No difference in risk was found for the remaining evaluated AEs: For LEV headache, dizziness, fatigue, nasopharyngitis, nausea, diarrhea, depression, insomnia, vomiting, influenza, pharyngitis, agitation, anxiety, rash, accidental injury, urinary tract infection, constipation, abdominal pain and thrombocytopenia and for BRV headache, nasopharyngitis, nausea, urinary tract infection, convulsion, irritability, insomnia and tremor. Figure 2 and 3 present the results of the meta-analyses for the most frequently reported events. More detailed information and forest plots for all evaluated AEs can be found in Figs. S3–S35. Funnel plot for evaluated AEs with at least ten studies can be found in Figs. S36–S45. Indications of publication bias were detected only for irritability associated with LEV.

**Fig. 2:**
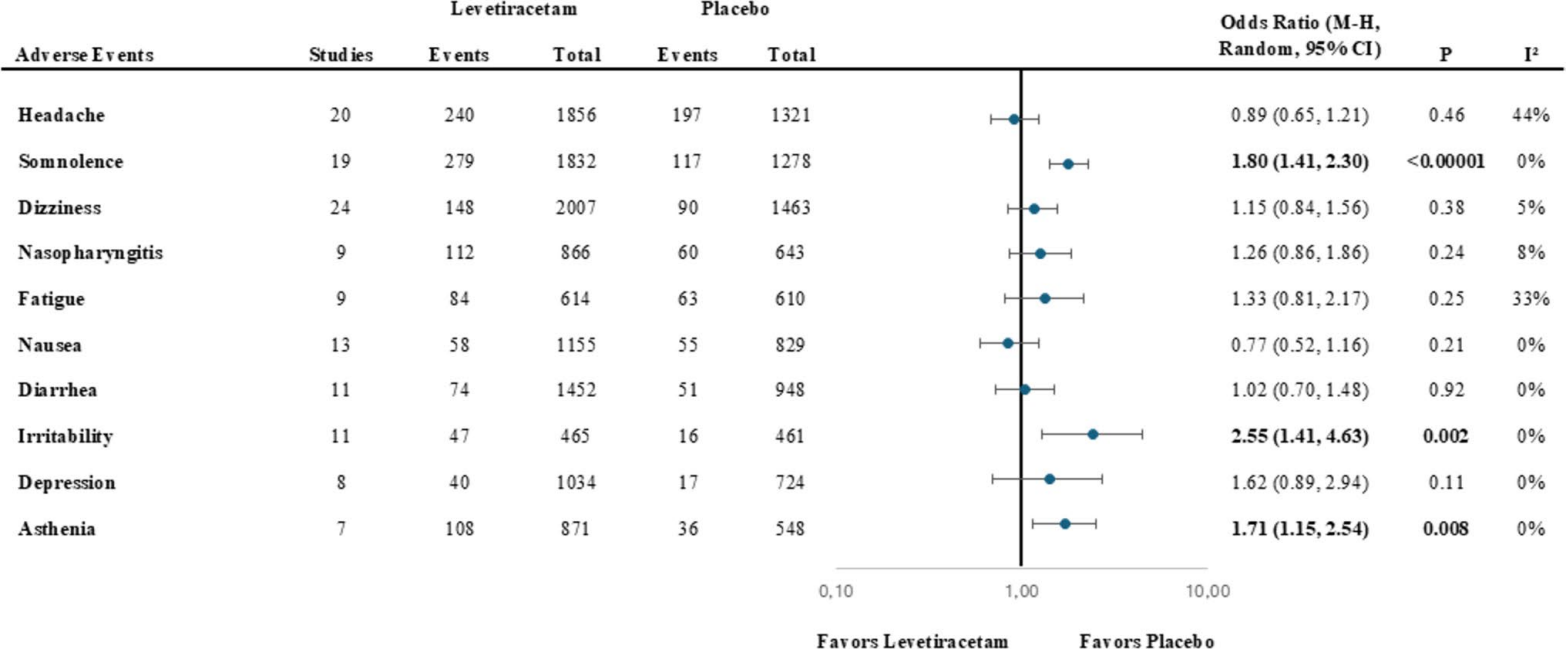
Forest plot for levetiracetam versus placebo for the ten most frequently reported adverse events M–H: Mantel–Haenszel, CI: confidence interval

**Fig. 3:**
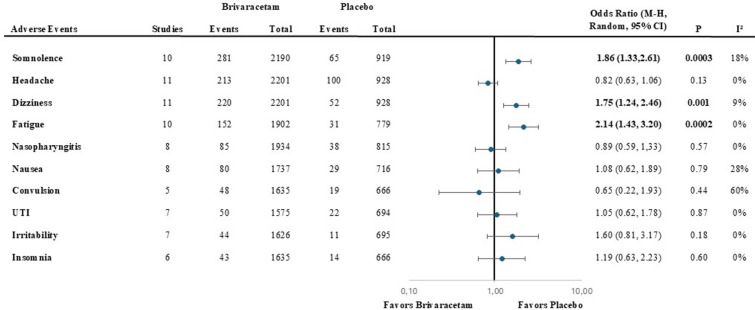
Forest plot for brivaracetam versus placebo for the ten most frequently reported adverse events M–H: Mantel–Haenszel, CI: confidence interval, UTI: Urinary tract infection

#### Sensitivity analyses

Viable sensitivity analyses are shown in Table S9. Overall, results of most sensitivity analyses supported the primary findings. Notably for LEV, the significant findings for somnolence and irritability lost significance when limited to actively monitored studies. Additionally, the effect for asthenia for LEV became non-significant when only studies with passive assessment were evaluated. The effect for dizziness for LEV became statistically significant when excluding all industry sponsored studies. All other sensitivity analyses did not yield any substantial deviations from primary results.

### Comparison of LEV and BRV

The descriptive comparison of AE frequencies across all administered doses indicated overall similar occurrence rates between LEV and BRV. However, twelve AEs showed nominal differences in aggregated frequencies of at least 2%, each with higher rates under LEV compared to BRV: aggression (4.17% vs. 1.19%), asthenia (6.37% vs. 1.35%), depressed mood (3.76% vs. 0.40%), depression (4.70% vs. 2.20%), fatigue (12.27% vs. 8.04%), headache (13.86% vs. 9.77%), irritability (6.24% vs. 2.51%), nasopharyngitis (11.18% vs. 4.37%), nervousness (3.16% vs. 0.70%), tremor (2.78% vs. 0.78%), thrombocytopenia (3.16% vs. 0.11%) and vertigo (5.17% vs. 2.77%) (see Tables S5–S6). While descriptive data suggested trends, they were not substantiated by indirect meta-analytic comparisons between. For the nine AEs where such comparisons were feasible, no statistically significant differences emerged between the two drugs (see Table S10). Finally, evidence from a single head-to-head trial directly comparing LEV and BRV reported no statistically significant differences in the frequencies of three AEs (asthenia/somnolence, headache, and behavioral changes) [33].

### Moderators of AE risks

#### Dose-AE relationship

**Fig. 4:**
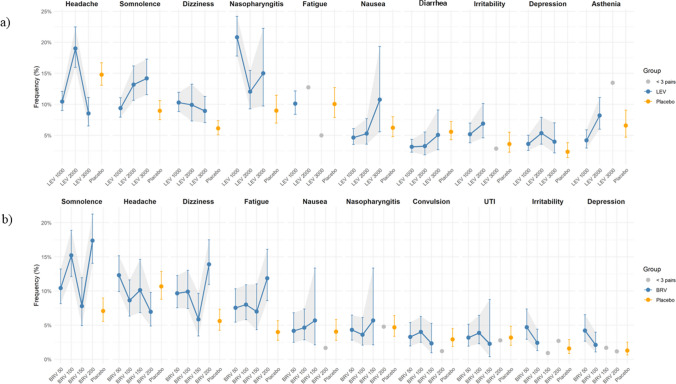
Frequencies of ten most frequently reported adverse events for levetiracetam (LEV) (**a**) and brivaracetam (BRV) (**b**) stratified by doses compared to placebo

The full overview of AE frequencies reported in  at least five studies stratified by different dosages for LEV and BRV can be found in Tables S5  and  S6 and for the ten most frequently reported AEs additionally in Fig. 4. For LEV, data from at least three studies per dose arm were available for seventeen AEs (Table S5, AEs marked in bold). A descriptive increase in frequency across rising dose levels was observed for diarrhea, nausea, somnolence, and pruritus. However, only somnolence consistently exceeded the mean frequency of the aggregated placebo arms at each dose level. For BRV, data from at least three studies per dose arm were available for four AEs (Table S6, AEs marked in bold). For none of these did AE frequencies increase with higher doses. Meta-regressions did not reveal any significant linear dose–response patterns in odds-ratios across all eligible analyses (Table S11). Finally, high-dose versus low-dose meta-analyses mostly supported these results. However, a higher risk of fatigue during BRV was found for higher compared to lower doses. All findings are compiled in Figs. S46–S58.

Ninety-five percent confidence intervals were calculated when at least three studies contributed data. UTI: urinary tract infection.

#### Subgroup analyses

Viable subgroup analyses were limited, particularly for BRV: Effects of age could only be investigated for 8 symptoms (LEV: 8; BRV: 0), effects of indication in 18 symptoms (LEV: 15; BRV: 3), effects of titration schemes in 21 symptoms (LEV: 11; BRV: 10) and effects of background medication in 15 symptoms (LEV: 15; BRV: 0). Only one statistically significant subgroup effect was observed indicating a higher risk for fatigue under LEV exposure in patients with other indications than epilepsy. For all other subgroup analyses no statistically significant effects were observed and the results are presented in Table S12.

## Discussion

The present study provides the most comprehensive synthesis to date of AE data from RCTs of LEV and BRV. Pooling evidence from 96 RCTs, this systematic review and meta-analysis characterizes the full spectrum of AEs and identifies three principal findings: (1) Across both compounds a wide range of AEs was reported, with symptoms such as headache, somnolence, dizziness, and fatigue occurring frequently. Yet, despite these high background rates, only a limited number of AEs were significantly associated with either LEV or BRV when compared to placebo. (2) In a descriptive comparison of LEV and BRV, AE frequencies were higher during LEV for 11 AEs, with a difference of at least 2%. However, indirect comparisons between LEV and BRV detected no significant differences in any evaluable AE, suggesting no substantial differences in the kind of AEs in their overall safety profiles. (3) Moderator analyses revealed very few consistent influences on AE risk. In general, no dose-AE associations were observed for the evaluable AEs. The only exception was fatigue, for which higher BRV doses were linked to a greater risk in a high- versus low-dose comparative meta-analysis. Notably, dose and titration regimen did not meaningfully affect the likelihood of AEs, while an indication-specific effect was identified for fatigue under LEV, where patients with epilepsy exhibited a lower risk compared to those treated for other conditions.

In relation to previous systematic evaluations, our findings show both overlap and refinement. Verrotti et al. reported that LEV was generally well tolerated, with somnolence, asthenia/fatigue, nervousness/irritability, dizziness, and nasopharyngitis occurring more frequently compared to placebo [11]. These results are largely in line with our synthesis, although the associations with dizziness and nasopharyngitis could not be replicated in our larger dataset, and we deliberately avoided aggregating distinct AE terms. Zhu et al. likewise described a favorable safety profile for BRV, with fatigue and dizziness more frequent under BRV compared to placebo, while back pain occurred less frequently [15]. The associations with fatigue and dizziness were reproduced in our analysis, and somnolence additionally emerged as a relevant AE. Back pain, however, could not be evaluated due to stricter inclusion criteria for quantitative synthesis. Consistent with both earlier reviews, our data confirm the absence of robust dose-AE relationships. Importantly, our study extends the prior findings by integrating evidence from a substantially larger and more recent body of RCTs, including unpublished data, and by systematically examining potential moderators of AE occurrence.

With regard to comparative safety, previous evidence on AE risks under LEV and BRV has been inconsistent. Observational studies suggested a lower risk of psychiatric symptoms with BRV [16, 21, 34]. In contrast, two earlier meta-analyses based on indirect comparisons of RCTs reported either no differences between compounds for the analyzable AEs [35] or a potentially increased risk of dizziness with BRV [17]. In our multimodal analysis, no clear differences in AE risks between the two substances could be established. The analysis for dizziness indicated a trend toward an increased risk with BRV compared with LEV, however, this finding did not reach statistical significance. The qualitative comparison suggests that LEV may be associated with a higher risk of several AEs including psychiatric or behavioral ones. A strength of the qualitative assessment relative to the quantitative approach is its incorporation of a larger data sample. However, this observation must be interpreted with caution; unlike formal meta-analytic comparisons, these qualitative trends lack statistical control for potential confounders, such as differences in baseline risks or trial designs, making them more susceptible to bias. Also, AE frequencies depend heavily on the AE assessment methods, which were in some cases insufficiently specified or not comparable across the underlying studies [36]. Consequently, these findings primarily support the targeted generation of hypotheses to guide future, more selective research.

Several methodological differences distinguish our work from previous ones which we consider strengths of the present study: First, our patient sample is large with 7145 patients exposed to LEV and 2549 to BRV owing to broad inclusion criteria. Notably, we deliberately included eligible studies that were not double-blind or not conducted in parallel-group designs to maximize dataset size and strengthen the descriptive evidence for AE frequencies [37]. In the same vein, we included trials across all indications. While this approach introduced clinical heterogeneity, it significantly increased the statistical power and the breadth of the AE dataset, facilitating the detection of less frequent events. Furthermore, this broad inclusion allowed us to investigate whether AEs cluster with specific disorders through pre-specified moderator analyses. Second, we incorporated unpublished datasets which substantially impacted the analyses by allowing finer differentiation between AEs and provided expanded evidence for rare and very rare AEs. Third, we applied multiple sensitivity analyses to test the robustness of our findings, which is particularly important given the substantial proportion of studies with indicators of industry sponsorship (LEV: 10, BRV: 10) that may report favorable safety outcomes [38]. Fourth, we adopted a comprehensive, three-pronged approach to evaluating dose dependencies by combining qualitative assessment, meta-regressions, and a meta-analytic high-dose versus low-dose comparisons to mitigate potential biases and sources of heterogeneity. Finally, we examined additional moderators, including patient’s age, indication for treatment, drug titration regimes, and the presence and type of background medication.

When interpreting these results, several limitations should be considered. First, many records applied thresholds in AE reporting. Such selective reporting may bias frequency estimates and underestimate rare or very rare events [39, 40]. Therefore, we contacted study authors and principal investigators and were able to include unpublished datasets to mitigate the risk. Second, a lack of standardized AE terminology may introduce heterogeneity, as similar symptoms may be labelled differently, and apparently identical AEs may in fact be defined or assessed inconsistently. To address this, we clustered similar AE terms in line with MedDRA guidance. Third, AE assessment methods were in several studies unspecified or followed a non-systematic approach. The methods being used have a meaningful impact on the detection of AEs [36]. Passive AE reporting, common in several LEV (37) and BRV (12) studies, can lead to underreporting of unprompted symptoms, whereas active assessment tends to detect higher AE rates. Although we attempted to address this through planned sensitivity analyses, feasibility constraints limited their scope. Furthermore, the generalizability of our findings to specific clinical subpopulations requires cautious interpretation. While this meta-analysis provides a robust safety estimate, the standardized framework of RCTs may not fully capture interindividual variability driven by biological and clinical factors: For instance, the use of broad diagnostic categories (e.g. “epilepsy” or “generalized epilepsy”) in trials may obscure syndrome-specific vulnerabilities [41]. Distinct epilepsy syndromes are associated with different psychiatric risk profiles: Focal epilepsies, particularly temporal lobe epilepsy, show higher rates of depression, anxiety and psychotic symptoms, likely reflecting limbic network dysfunction, whereas generalized epilepsies, such as juvenile myoclonic epilepsy, are more commonly linked to mood and anxiety disorders [42]. Notably, elevated trait impulsivity in juvenile myoclonic epilepsy has been associated with an increased risk of psychiatric adverse outcomes under LEV treatment [43]. Emerging pharmacogenomic data further suggest that genetic variation, including polymorphisms affecting dopaminergic pathways [44], may modulate susceptibility to psychiatric adverse events, an important level of granularity not captured in current RCT datasets. For BRV, which is metabolized via CYP2C19, poor metabolizers may experience greater exposure and, consequently, a higher risk of adverse events [45]. Age represents another potential moderator of tolerability. Although our meta-analysis synthesized data across the lifespan, only a limited number of included RCTs focused exclusively on pediatric populations (LEV: 16; BRV: 1), and age-stratified analyses remained limited. While efficacy findings are often extrapolated from adults to children, safety and tolerability may differ substantially due to neurodevelopmental factors, psychiatric comorbidity patterns, pharmacokinetics, and the behavioral manifestation of AEs [46]. Clinical implications for pediatric patients should therefore be interpreted with caution. Finally, our findings may not extend to acute or neurocritical care settings, which were excluded due to short treatment duration. In these contexts, altered pharmacokinetics, renal function, and acute illness may substantially modify adverse event risk and should inform clinical decision-making [47].

The findings of our meta-analysis entail several important clinical implications. First, they confirm that both LEV and BRV are generally well tolerated and exhibit no major differences in the kind of AEs in their safety profiles. This reinforces earlier evidence and substantiates the established role of both agents in the pharmacological management of epilepsy [48].

Importantly, our analyses did not reveal a robust dose–response relationship for AEs, indicating that higher doses can be employed when necessary for seizure control without a disproportionate increase in AE burden. An exception may be fatigue associated with BRV therapy, for which comparisons of high versus low dosing regimens demonstrate an increased risk at higher doses. In addition, concomitant administration of another ASM, which could plausibly act as a moderator of AE risk, did not alter the likelihood of AEs for LEV. For BRV, however, such analyses could not be conducted due to insufficient data.

As for choosing between LEV and BRV, our results indicate that both compounds can be considered safe options for clinical use, with tolerability unlikely to be a decisive factor in therapeutic decision-making. Instead, treatment choice should be informed primarily by individual patient characteristics, clinical context, and previous treatment experience. A limitation remains in the domain of psychiatric AEs, where the available RCT evidence was limited for comparative analysis. In this regard, observational studies suggesting a lower risk with BRV provide valuable complementary insights.

While our review advances the understanding of LEV and BRV safety, it also highlights several areas where further research is needed. One clear recommendation is for future clinical trials (and regulatory reporting) to adopt more standardized and comprehensive methods of AE collection. Inconsistencies in how AEs are defined and reported across studies contribute to heterogeneity and make comparisons challenging. Employing uniform terminology (for example, leveraging MedDRA classifications for symptom clustering) and active surveillance techniques (such as structured questionnaires or regular side effect checklists) in trials would improve the detection of AEs, especially those that might otherwise go unreported. Second, the findings from our qualitative analysis serve as hypothesis-generating, as they suggest potential associations between LEV and BRV based on a larger dataset that could not be replicated in our more restrictive quantitative sample. Consequently, future studies are needed to confirm or refute these observations. Third, the field would benefit from more direct comparative studies between LEV and BRV. To date, most evidence regarding their relative safety has come from observational studies or indirect comparisons of data gained from RCTs. The single head-to-head trial included in this analysis was limited by a relatively small sample size [33]. Finally, long-term pharmacovigilance and real-world studies should continue to complement RCT data, as they are better suited to capture rare or delayed AEs and overall tolerability in unselected populations. Encouraging the publication of unpublished trial data and results, as we have done by including several unpublished datasets, is another step that future research efforts should take to ensure the most complete evidence base. In sum, future research should strive not only to fill the knowledge gaps identified, but also to refine the methodology of how we study drug safety, thereby enabling clinicians to make even more informed decisions.

## Conclusion

In summary, this systematic review and meta-analysis provides the most comprehensive evidence to date on the safety of LEV and BRV. Both agents are generally well tolerated and exhibit comparable overall AE profiles, with only a limited number of specific AEs significantly associated with treatment. No robust dose–response relationships were observed, and moderator effects were minimal. Although descriptive analysis of AE frequencies indicated a higher incidence of certain AEs for LEV, particularly psychiatric ones like irritability and aggression, these observations remained exploratory as they could not be corroborated through direct and indirect comparisons. These results support the continued use of both LEV and BRV as safe therapeutic options in epilepsy. Future research should focus on standardized and active AE assessment, direct comparative trials, and long-term real-world data to refine our understanding of psychiatric and rare AEs.

## Supplementary Information

Below is the link to the electronic supplementary material.

Supplementary file1 (DOCX 3924 KB)

## Data Availability

The data underlying will be shared on reasonable request to the corresponding author. This publication is based on research using data from data contributor UCB that has been made available through Vivli, Inc. Vivli has not contributed to or approved, and is not in any way responsible for, the contents of this publication.

## Declarations

### Clinical trial registration

Registered in PROSPERO: CRD42023491050 (6.12.2023) and CRD420251003207 (3.3.2025).
