# Supplementary material for: Reassessing the adverse event profiles of levetiracetam and brivaracetam: a systematic review and meta-analysis
Source: J Neurol. 2026 Mar 13;273(3):204. doi: 10.1007/s00415-026-13746-9 (PMC13437759; doi:10.1007/s00415-026-13746-9)
Supplement: Supplementary file 1 [file 415_2026_13746_MOESM1_ESM.docx]

Supplementary Materials to

**Reassessing the adverse event profiles of Levetiracetam and Brivaracetam: A systematic review and meta-analysis**

Emma Fröling^1^ (ORCID: 0009-0000-6501-3361), Mariel Morales Sahm^1^ (ORCID: 0009-0005-8880-0380), Marcel Schmude² (ORCID: 0000-0002-7519-1724), Charlotte P. Assies^3^, Michael Wittenberg^4^ (ORCID: 0000-0003-4526-1856), Felix Rosenow^5^ (ORCID: 0000-0002-3989-7471), Felix Bermpohl^1^ (ORCID: 0000-0002-3260-6328), and Thomas G. Riemer^1,2*^ (ORCID: 0000-0002-8068-3192)

^1^ Charité – Universitätsmedizin Berlin, corporate member of Freie Universität Berlin, Humboldt-Universität zu Berlin, and Berlin Institute of Health, Department of Psychiatry and Psychotherapy, 10115 Berlin, Germany

² Charité – Universitätsmedizin Berlin, corporate member of Freie Universität Berlin, Humboldt-Universität zu Berlin, and Berlin Institute of Health, Institute of Clinical Pharmacology and Toxicology, 10117 Berlin, Germany

^3^ Heidelberg University, Institute of Psychology, 69117 Heidelberg, Germany

^4^ Marburg University, Faculty of Medicine, Coordinating Center of Clinical Trials, Department Marburg, 35043 Marburg, Germany

^5^ Goethe-Universität Frankfurt, Epilepsy Center Frankfurt Rhine-Main, Department of Neurology, Universitätsmedizin Frankfurt, 60590 Frankfurt a. M., Germany

* Corresponding author

Emma Fröling: [emma.froeling@charite.de](mailto:emma.froeling@charite.de)

Mariel Morales Sahm: [mariel.morales-sahm@charite.de](mailto:mariel.morales-sahm@charite.de)

Marcel Schmude: [marcel.schmude@charite.de](mailto:marcel.schmude@charite.de)

Charlotte P. Assies: [charlotte.assies@stud.uni-heidelberg.de](mailto:charlotte.assies@stud.uni-heidelberg.de)

Michael Wittenberg: [michael.wittenberg@kks.uni-marburg.de](mailto:michael.wittenberg@kks.uni-marburg.de)

Felix Rosenow: [rosenow@med.uni-frankfurt.de](mailto:rosenow@med.uni-frankfurt.de)

Felix Bermpohl: [felix.bermpohl@charite.de](mailto:felix.bermpohl@charite.de)

Thomas G. Riemer: [thomas.riemer@charite.de](mailto:thomas.riemer@charite.de)

Journal of Neurology

**Table of Contents**

[**Table S1 Inclusion and exclusion criteria**	4](#_Toc222677577)

[**Table S2 Rationale for sensitivity analyses**	5](#_Toc222677578)

[**Table S3 Overview of included studies - LEV**	6](#_Toc222677579)

[**Table S4 Overview of included studies - BRV**	13](#_Toc222677580)

[**Fig. S1 Overview of results of Risk of Bias rating – LEV**	15](#_Toc222677581)

[**Fig. S2 Overview of results of Risk of Bias rating – BRV**	15](#_Toc222677582)

[**Evaluation of AE profiles of LEV and BRV**	16](#_Toc222677583)

[**Table S5 Frequencies of AEs reported in at least 5 studies - LEV**	16](#_Toc222677584)

[**Table S6 Frequencies of AEs reported in at least 5 studies – BRV**	24](#_Toc222677585)

[**Table S7 All reported AEs in less than 5 studies- LEV**	27](#_Toc222677586)

[**Table S8 All reported AEs in less than 5 studies – BRV**	35](#_Toc222677587)

[**Fig. S3 Forest plot for headache - LEV vs. PLC**	36](#_Toc222677588)

[**Fig. S4 Forest plot for somnolence – LEV vs. PLC**	37](#_Toc222677589)

[**Fig. S5 Forest plot for dizziness – LEV vs. PLC**	38](#_Toc222677590)

[**Fig. S6 Forest plot for fatigue – LEV vs. PLC**	39](#_Toc222677591)

[**Fig. S7 Forest plot for nasopharyngitis – LEV vs. PLC**	40](#_Toc222677592)

[**Fig. S8 Forest plot for nausea – LEV vs. PLC**	41](#_Toc222677593)

[**Fig. S9 Forest plot for diarrhea – LEV vs. PLC**	42](#_Toc222677594)

[**Fig. S10 Forest plot for irritability – LEV vs. PLC**	43](#_Toc222677595)

[**Fig. S11 Forest plot for asthenia – LEV vs. PLC**	44](#_Toc222677596)

[**Fig. S12 Forest plot for depression – LEV vs. PLC**	45](#_Toc222677597)

[**Fig. S13 Forest plot for insomnia – LEV vs. PLC**	46](#_Toc222677598)

[**Fig. S14 Forest plot for vomiting – LEV vs. PLC**	47](#_Toc222677599)

[**Fig. S15 Forest plot for influenza – LEV vs. PLC**	48](#_Toc222677600)

[**Fig. S16 Forest plot for pharyngitis – LEV vs. PLC**	49](#_Toc222677601)

[**Fig. S17 Forest plot for agitation – LEV vs. PLC**	50](#_Toc222677602)

[**Fig. S18 Forest plot for anxiety – LEV vs. PLC**	51](#_Toc222677603)

[**Fig. S19 Forest plot for rash – LEV vs. PLC**	52](#_Toc222677604)

[**Fig. S20 Forest plot for accidental injury – LEV vs. PLC**	53](#_Toc222677605)

[**Fig. S21 Forest plot for urinary tract infection – LEV vs. PLC**	54](#_Toc222677606)

[**Fig. S22 Forest plot for constipation – LEV vs. PLC**	55](#_Toc222677607)

[**Fig. S23 Forest plot for abdominal pain – LEV vs. PLC**	56](#_Toc222677608)

[**Fig. S24 Forest plot for thrombocytopenia – LEV vs. PLC**	57](#_Toc222677609)

[**Fig. S25 Forest plot for headache – BRV vs. PLC**	58](#_Toc222677610)

[**Fig. S26 Forest plot for somnolence – BRV vs. PLC**	59](#_Toc222677611)

[**Fig. S27 Forest plot for dizziness – BRV vs. PLC**	60](#_Toc222677612)

[**Fig. S28 Forest plot for fatigue – BRV vs. PLC**	61](#_Toc222677613)

[**Fig. S29 Forest plot for nasopharyngitis – BRV vs. PLC**	62](#_Toc222677614)

[**Fig. S30 Forest plot for nausea – BRV vs. PLC**	63](#_Toc222677615)

[**Fig. S31 Forest plot for urinary tract infection – BRV vs. PLC**	64](#_Toc222677616)

[**Fig. S32 Forest plot for convulsion – BRV vs. PLC**	65](#_Toc222677617)

[**Fig. S33 Forest plot for irritability – BRV vs. PLC**	66](#_Toc222677618)

[**Fig. S34 Forest plot for insomnia – BRV vs. PLC**	67](#_Toc222677619)

[**Fig. S35 Forest plot for tremor – BRV vs. PLC**	68](#_Toc222677620)

[**Fig. S36 Funnel plot for headache – LEV vs. PLC**	69](#_Toc222677621)

[**Fig. S37 Funnel plot for somnolence – LEV vs. PLC**	70](#_Toc222677622)

[**Fig. S38 Funnel plot for dizziness – LEV vs. PLC**	71](#_Toc222677623)

[**Fig. S39 Funnel plot for nausea – LEV vs. PLC**	72](#_Toc222677624)

[**Fig. S40 Funnel plot for diarrhea - LEV vs. PLC**	73](#_Toc222677625)

[**Fig. S41 Funnel plot for irritability – LEV vs. PLC**	74](#_Toc222677626)

[**Fig. S42 Funnel plot for insomnia – LEV vs. PLC**	75](#_Toc222677627)

[**Fig. S43 Funnel plot for somnolence – BRV vs. PLC**	76](#_Toc222677628)

[**Fig. S44 Funnel plot for dizziness – BRV vs. PLC**	77](#_Toc222677629)

[**Fig. S45 Funnel plot for fatigue – BRV vs. PLC**	78](#_Toc222677630)

[**Table S9 Results of sensitivity analyses**	79](#_Toc222677631)

[**Table S10 Results of comparison – LEV vs. BRV**	85](#_Toc222677632)

[**Table S11 P-values of meta regressions for evaluated AEs for LEV and BRV**	86](#_Toc222677633)

[**Fig. S46 Forest plot high dose vs low dose for headache – LEV**	87](#_Toc222677634)

[**Fig. S47 Forest plot high dose vs low dose for somnolence – LEV**	88](#_Toc222677635)

[**Fig. S48 Forest plot high dose vs low dose for dizziness – LEV**	89](#_Toc222677636)

[**Fig. S49 Forest plot high dose vs low dose for headache – BRV**	90](#_Toc222677637)

[**Fig. S50 Forest plot high dose vs low dose for somnolence – BRV**	91](#_Toc222677638)

[**Fig. S51 Forest plot high dose vs low dose for dizziness – BRV**	92](#_Toc222677639)

[**Fig. S52 Forest plot high vs low dose for fatigue – BRV**	93](#_Toc222677640)

[**Fig. S53 Forest plot high dose vs low dose for nasopharyngitis – BRV**	94](#_Toc222677641)

[**Fig. S54 Forest plot high dose vs low dose for nausea – BRV**	95](#_Toc222677642)

[**Fig. S55 Forest plot high dose vs low dose for convulsion – BRV**	96](#_Toc222677643)

[**Fig. S56 Forest plot high dose vs low dose for irritability – BRV**	97](#_Toc222677644)

[**Fig. S57 Forest plot high dose vs low dose for urinary tract infection – BRV**	98](#_Toc222677645)

[**Fig. S58 Forest plot high dose vs low dose for insomnia – BRV**	99](#_Toc222677646)

[**Table S12 Results of subgroup analyses**	100](#_Toc222677647)

## **Table S1 Inclusion and exclusion criteria**

|  | **Inclusion criteria** | **Exclusion criteria** |
| --- | --- | --- |
| Population | Human | Non-human subjects |
| Intervention | Minimum one arm with Levetiracetam or Brivaracetam monotherapy, systematically administered ≥ 14 consecutive days | No Levetiracetam or brivaracetam therapy, only combination therapy regimens, treatment duration < 14 consecutive days |
| Comparator | Placebo or active comparator (including different dosing regimens of Levetiracetam or Brivaracetam monotherapies) | No control group |
| Outcome(s) | Report of at least 1 adverse event in any group expressed as frequencies of affected patients (including 0) | No report of any adverse events or no indication of concrete frequencies, but other types of information |
| Study Design | Prospective, clinical intervention studies* | All other study types (e.g. retrospective studies, observational studies) |
| Other | - | Data implausible, duplicate data, record unavailable |

*We included pooled data when the constituent studies met the inclusion criteria but individual AE data were unavailable.

## **Table S2 Rationale for sensitivity analyses**

| **Analysis** | **Justification for the analysis** |
| --- | --- |
| Exclusion of the study with the greatest weight | Results of the study with the greatest weight might have introduced a bias to the analysis which can lead to disproportionately effects driven by a single study. |
| Restricted analysis to studies with a treatment duration > 8 weeks | Some adverse events may manifest only after a prolonged exposure to treatment. |
| Exclusion of studies with a risk of bias rated as „high risk“ | Studies rated as having a „high risk“ of bias may misestimate specific adverse events due to methodological limitations, such as unblinding. |
| Restricted analysis to studies with a stated active assessment of adverse events | Active assessment of adverse events can lead to an overestimation of adverse events due to priming effects, where participants may become aware of specific symptoms. |
| Restricted analysis to studies with a stated passive assessment of adverse events | Passive assessment of adverse events follows more naturalistic reporting methods but may result in underreporting of specific symptoms. |
| Exclusion industry-sponsored studies | Industry sponsored studies may be subject to reporting bias, potentially favouring more favourable safety profiles. |
| Exclusion of pooled data | Inclusion of pooled data can artificially inflate study weights and thereby bias comparative effect estimates. |
| Exclusion of unpublished data | Unpublished studies may differ systematically from published studies in methodological quality and reporting transparency, potentially introducing bias into the overall findings. |
|  |  |
|  |  |

## **Table S3 Overview of included studies - LEV**

| **Author** | **ClinicalTrials.gov Identifier** | **Study design** | **Blinding** | **Treatment duration** | **Target dose** | **Exposed patients (LEV)** | **Exposed patients (PLC)** | **Exposed patients (active comparator)** | **AE threshold** | **Risk of Bias** | **Comment** |
| --- | --- | --- | --- | --- | --- | --- | --- | --- | --- | --- | --- |
| Ahadi P et al. (2020) | - | Parallel-group | Open-label | 6 months | 25-30 mg/kg/day | 46 | - | CAR: 46 | Not specified | High risk |  |
| Akter N et al. (2018) | - | Parallel-group | Open-label | 1 year | Not specified | 50 | - | PB: 68 | Not specified | High risk |  |
| Archna et al. (2022) | NCT04172311 | Parallel-group | Open-label | 12 weeks | 60 mg/kg/d | 50 | - | MAD: 51 | Not specified | High risk |  |
| Ben-Menachem E et al. (2000) | - | Parallel-group | Double-blind | 12 weeks | 3000 mg/d | 181 | 105 | - | 5% | High risk |  |
| Beran RG et al. (2011) | - | Cross-over | Double-blind | 11 weeks | 3000 mg/d | 89 | 88 | - | Not specified | Some concerns |  |
| Berkovic SF et al. (2007) | NCT00150748; NCT00160550 | Parallel-group | Double-blind | 24 weeks | 3000 mg/d | 79 | 84 | - | 5% | Low risk |  |
| Betts T et al. (2000) | - | Parallel-group | Double-blind | 24 weeks | 2000, 3000, 4000 mg/d | 80 | 39 | - | 10% | High risk |  |
| Brodie MJ et al. (2007) | NCT00150787, NCT00150735 | Parallel-group | Double-blind | 26 weeks | 1000 - 3000 mg/d | 285 | - | CAR: 291 | most common | Low risk |  |
| Cereghino JJ et al. (2000) | - | Parallel-group | Double-blind | 26 weeks | 1000, 2000, 3000 mg/d | 199 | 95 | - | 10% | Some concerns |  |
| Chung S et al. (2012) | NCT00419094 | Parallel-group | Triple-blinded | 18 weeks | 1000, 2000 mg/d | 228 | - | - | 5% | Low risk |  |
| Consoli D et al. (2012) | - | Parallel-group | Open-label | 54 weeks | 1000 mg/d | 52 | - | CAR: 54 | Not specified | High risk |  |
| Coppola G et al. (2007) | - | Parallel-group | Open-label | 12-24 months | 20 mg/kg/d | 21 | - | OXA: 18 | Not specified | High risk |  |
| Cumbo et al. (2010) | - | Parallel-group | Not specified | 12 months | Not specified | 35 | - | LTG: 25 PB: 23 | 0% | High risk |  |
| Daneshyar S et al. (2022) | - | Parallel-group | Double-blind | 12 months | Not specified | 31 | - | LTG: 30 VALP: 41 | Top reported events | Some concerns |  |
| de Tommaso M et al. (2007; 2008) | - | Parallel-group | Double-blind | 8 weeks | 1000 mg/d | 15 | 15 | TPM: 15 | Not specified | Some concerns | 2 paper focusing on 1 trial, Aes the same in both paper |
| Elble RJ et al. (2007) | - | Cross-over | Double-blind | 9 weeks | Not specified | 15 | 15 | - | Not specified | Some concerns |  |
| Faghihjouibari M et al. (2023) | - | Parallel-group | Not specified | 95 days | 1000 mg/d | 39 | - | PHT: 40 | Not specified | Some concerns |  |
| Fattore C et al. (2011) | - | Parallel-group | Double-blind | 2 weeks | Not specified | 38 | 21 | - | Not specified | High risk |  |
| Fertig KB (2012) | NCT00970814 | Parallel-group | Double-blind | 16 weeks | 2000 mg/d | 64 | 66 | - | 5% | Some concerns |  |
| Finnerup NB et al. (2009) | - | Cross-over | Double-blind | 5 weeks | Not specified | 34 | 32 | - | Not specified | High risk |  |
| Giussani G et al. (2025) |  | Parallel-group | Single-blind | 12 months | 500 and 1000 mg/d | 23 | - | - | 0% | High risk |  |
| Glauser TA et al. (2006) | - | Parallel-group | Double-blind | 14 weeks | Not specified | 101 | 97 | - | 1% | Low risk |  |
| Handforth A et al. (2004) | - | Cross-over | Double-blind | 6 weeks | 3000 mg/d | 7 | 2 | - | Not specified | Some concerns |  |
| Hedderick EF et al. (2009) | - | Cross-over | Double-blind | 6 weeks | Not specified | 10 | - | CLO: 10 | Not specified | Some concerns |  |
| Holbech JV et al. (2011) | NCT00286260 | Cross-over | Double-blind | 6 weeks | 3000 mg/d | 37 | 37 | - | Not specified | High risk |  |
| Inoue Y et al. (2015) | NCT00280696 | Parallel-group | Double-blind | 16 weeks | 500, 1000, 2000, 3000 mg/d | 281 | 70 | - | 5% | Low risk |  |
| Jaiswal BK et al. (2023) | - | Parallel-group | Open-label | 15-30 days | Not specified | 15 | - | LCM: 10 | Not specified | High risk |  |
| Jung DE et al. (2015) | NCT02208492 | Parallel-group | Open-label | 52 weeks | Not specified | 57 | - | CAR: 64 | 0% | High risk |  |
| Jungehulsing GJ et al. (2013) | - | Cross-over | Double-blind | 8 weeks | Not specified | 40 | 35 | - | All AEs more frequent in LEV group | Some concerns |  |
| Kim JH et al. (2017) | NCT01498822 | Parallel-group | Open-label | 48 weeks | 1000 mg/d | 173 | - | OXA: 174 | 5% | High risk |  |
| Knapp CM et al. (2015) | NCT00862563 | Parallel-group | Double-blind | 14 weeks | 2000 mg/d | 21 | 24 | TPM: 21 ZONI: 19 | 5% | Some concerns |  |
| Labiner DM et al. (2009) | - | Parallel-group | Double-blind | 20 weeks | 2000 mg/d | 136 | - | LTG: 132 | 0% | Low risk |  |
| Latif Z et al. (2025) | - | Parallel-group | No information | 6 months | - | 100 | - | VALP: 100 | 0% | Some concerns |  |
| Lee SK et al. (2019) | NCT01229735 | Parallel-group | Open-label | 52 weeks | 1000 mg/d | 177 | - | TPM: 166 | 5% | High risk | - |
| Levisohn PM et al. (2009) | NCT00105040 | Parallel-group | Double-blind | 12 weeks | Not specified | 64 | 34 | - | 5% | Some concerns |  |
| Liang S et al. (2017) | - | Parallel-group | Not specified | 24 weeks | Not specified | 96 | 91 | - | Not specified | High risk |  |
| Marson AG et al. (2022) | - | Parallel-group | Open-label | 6 weeks | 1000 mg/d | 258 330 | - - | VALP: 257 LTG: 328 ZON: 324 | 0% | High risk | 2 studies in 1 paper published |
| Mattes JA et al. (2008) | - | Parallel-group | Double-blind | 10 weeks | 1000 - 3000 mg/d | 20 | 20 | - | AEs in more than 1 LEV treated patient | Some concerns |  |
| Mohs R et al. (2023) | - | Parallel-group | Double-blind | 78 weeks | 220 mg/d | 78 | 83 | - | treatment-related | Some concerns |  |
| Montazerlotfelahi H et al. (2019) | - | Parallel-group | Double-blind | 12 weeks | Not specified | 34 | 34 | - | Not specified | Some concerns |  |
| Montazerlotfelahi H et al. (2024) | - | Parallel-group | Double-blind | 24 weeks | Not specified | 60 | - | CAR: 60 | most common | High risk |  |
| Noachtar S et al. (2008) | NCT00150774 | Parallel-group | Double-blind | 22 weeks | 3000 mg/d | 60 | 60 | - | 5% | Some concerns |  |
| Pandya et al. (2025) | - | Parallel-group | Single-blind | 3 months | - | 50 | - | BRV: 50 | most common | High risk |  |
| Peltola J et al. (2009) | NCT00368069 | Parallel-group | Double-blind | 12 weeks | 1000 mg/d | 77 | 79 | - | 5% | High risk |  |
| Peter-Derex L et al. (2021) | NCT02631759 | Parallel-group | Double-blind | 6 weeks | 1000 mg/d | 23 | 25 | - | 5% | Some concerns |  |
| Pohlmann-Eden B et al. (2016) | NCT00175903 | Parallel-group | Open-label | 52 weeks | 1000 mg/d | 151 | - | CAR: 103 VALP: 49 | 5% | High risk | Subgroup analysis of KOMET study only Contusion additionally reported and analyzed in here |
| Ragueneau-Majlessi I et al. (2002) | - | Cross-over | Double-blind | 21 days | 1000 mg/d | 18 | 18 | - | at least in 2 subjects | Some concerns |  |
| Rahman M et al. (2023) | NCT02334722 | Parallel-group | Not specified | 6 weeks | 1000 mg/d | 41 | - | - | 5% | High risk |  |
| Rosenfeld WE et al. (2009) | NCT00150774; NCT00160550 | Parallel-group | Double-blind | 16-24 weeks | 3000 mg/d or 60 mg/kg/d | 113 | 128 | - | 5% | Some concerns |  |
| Rosenow F et al. (2012) | NCT00242606 | Parallel-group | Open-label | 26 weeks | 2000 mg/d | 204 | - | LTG: 201 | Most common AEs | High risk |  |
| Rossetti AO et al. (2014) | NCT00629889 | Parallel-group | Open-label | 12 months | 3000 mg/d | 25 | - | PRG: 27 | Not specified | High risk |  |
| Rossi S et al. (2009) | - | Parallel-group | Single-blind | 12 weeks | 3000 mg/d | 12 | 8 | - | Not specified | Some concerns |  |
| Saricicek A et al. (2011) | NCT00566150 | Parallel-group | Double-blind | 6 weeks | 500-3000 mg/d | 19 | 16 | - | Not specified | Some concerns |  |
| Schoenberg MR et al. (2017) | NCT00221988 | Cross-over | Double-blind | 5 weeks | 1000 mg/d | 20 | 20 | - | Not specified | Some concerns |  |
| Shorvon SD et al. (2000) | - | Cross-over* | Double-blind | 16 weeks | 1000, 2000 mg/d | 212 | 112 | - | 5% | Low risk | * AE report separate for each phase |
| Stein MB et al. (2010) | NCT00612859 | Parallel-group | Double-blind | 12 weeks | Not specified | 110 | 106 | - | Most commonly reported | Low risk |  |
| Suresh SH et al (2015) | - | Parallel-group | Open-label | 26 weeks | Not specified | 30 | - | CAR: 30 | Not specified | High risk |  |
| Trinka E et al. (2013) | NCT00175903 | Parallel-group | Open-label | 52 weeks | 1000 mg/d | 835 | - | CAR: 499  VALP: 342 | 5% | High risk |  |
| Tsai JJ et al. (2006) | - | Parallel-group | Double-blind | 14 weeks | 2000 mg/d | 47 | 47 | - | 5% | Some concerns |  |
| Verma A et al. (2013) | - | Parallel-group | Not specified | 4 months | 1000 mg/d | 32 | 33 | - | Not specified | Low risk |  |
| Vilholm OJ et al. (2008) | NCT00262262 | Cross-over | Double-blind | 4 weeks | 3000 mg/d | 25 | 25 | - | Not specified | High risk |  |
| Vossel K et al. (2021) | NCT02002819 | Cross-over | Double-blind | 4 weeks | 300 mg/d | 33 | 32 | - | 0% | High risk |  |
| Wang M et al. (2017) | - | Parallel-group | Rater/Investigator-blinded | 6 months | 60 mg/kg/d | 35 | - | EDT: 35 | Not specified | High risk |  |
| Wasserman S et al. (2006) | - | Parallel-group | Double-blind | 10 weeks | Not specified | 10 | 10 | - | Not specified | High risk |  |
| Werhahn KJ et al. (2015) | NCT00438451 | Parallel-group | Double-blind | 58 weeks | 1000 mg/d | 122 | - | CAR: 121  LTG: 117 | 5% | Low risk |  |
| Wolz M et al. (2010) | NCT00307450 | Parallel-group | Double-blind | 13 weeks | Not specified | 17 | 14 | - | AEs occurred in at least 2 patients | Some concerns |  |
| Woods SW et al. (2008) | NCT00291213 | Parallel-group | Double-blind | 12 weeks | Not specified | 25 | 25 | - | Any increase from baseline at any time | Some concerns |  |
| Wu L et al. (2018) | NCT01228747 | Parallel-group | Triple-blind | 28 weeks | 1000, 2000, 3000 mg/d | 126 | 125 | - | 5% | Low risk |  |
| Wu T et al. (2018) | - | Parallel-group | Double-blind | 12 weeks | 1000 mg/d | 67 | - | - | Not specified | High risk |  |
| Wu XY et al. (2009) | NCT00152373 | Parallel-group | Double-blind | 16 weeks | 3000 mg/d | 103 | 103 | - | 5% | Low risk |  |
| Xiao Z et al. (2009) | - | Parallel-group | Double-blind | 16 weeks | 3000 mg/d | 28 | 28 | - | 5% | Low risk |  |
| Zaccara G et al. (2014) | NCT00537238 | Parallel-group | Double-blind | 16 weeks | 1000, 2000, 3000 mg/d | 255 | - | PRG: 254 | 5% | Low risk |  |
|  | NCT00916149 | Parallel-group | Open-label | 8 weeks | 3000 mg/d | 8 | 12 | - | 0% | High risk |  |
|  | NCT01506882 | Parallel-group | Open-label | 26 weeks | 1000, 2000, 3000 mg/d | 71 | - | - | 0% | High risk |  |
|  | NCT00577005 | Parallel-group | Triple-blinded | 12 weeks | 3000 mg/d | 16 | - | METH: 12 | 1% | Some concerns |  |
|  | NCT02201251 | Parallel-group | Open-label | 1 year | Not specified | 35 | - | TPM: 28 | 2% | High risk |  |
|  | NCT01954121 | Parallel-group | Open-label | 30 weeks | 1000 mg/d | 218 | - | CAR: 215 | 5% | High risk |  |
|  | NCT01044758 | Cross-over | Double-blind | 2 weeks | 125, 250, 500 mg/d | 69 | 69 | - | 5% | Some concerns |  |
|  | NCT03436433 | Parallel-group | Open-label | 30 days | 2000 mg/d | 2 | 1 | LCM: 1 | 0% | High risk |  |
|  | NCT00774306 | Parallel-group | Open-label | 8 weeks | 1000-1500 mg/d | 16 | 7 | PHT: 24  VALP: 5 | 5% | High risk |  |
|  | NCT03461861 | Cross-over | Double-blind | 2 weeks | 220 mg/d | 26 | 26 | - | 3% | High risk |  |
|  | NCT02707965 | Cross-over | Double-blind | 16 weeks | Not specified | 5 | - | CAR: 4  LTG: 4 TPM: 5 VALP: 2  ZONI: 1 | 5% | High risk |  |

## **Table S4 Overview of included studies - BRV**

| **Author** | **ClinicalTrials.gov Identifier** | **Study characteristics** | **Treatment duration** | **Exposed patients (BRV)** | **Exposed patients (PLC)** | **AE threshold** | **Risk of Bias** | **Comment** |
| --- | --- | --- | --- | --- | --- | --- | --- | --- |
| Arnold S et al. (2018) | NCT00698581 | Phase 3, double-blind, randomized, historical-controlled | 16 weeks | 88 | - | 5% | Low |  |
| Arnold S et al. (2018) | NCT00699283 | Phase 3, double-blind, randomized, historical-controlled | 16 weeks | 62 | - | 5% | Low |  |
| Biton V et al. (2014) | NCT00464269 | Phase 3, double-blind, randomized, placebo-controlled | 12 weeks | 298 | 98 | 5% | Low |  |
| Inoue Y et al. (2024) | NCT03083665 | Phase 3, double-blind, randomized, placebo-controlled | 12 weeks | 299 | 149 | 3% | Low |  |
| Kälviäinen R et al. (2016) | NCT00368251 | Phase 3, double-blind, randomized, placebo-controlled | 14 weeks | 38 | 18 | None | Some concerns |  |
| Kälviäinen R et al. (2016) | NCT00357669 | Phase 3, double-blind, randomized, placebo-controlled | 14 weeks | 34 | 16 | None | Some concerns |  |
| Klein P et al. (2015) | NCT01261325 | Phase 3, double-blind, randomized, placebo-controlled | 12 weeks | 503 | 261 | 5% | Some concerns |  |
| Kwan P et al. (2014) | NCT00504881 | Phase 3, double-blind, randomized, placebo-controlled | 16 weeks | 359 | 121 | 5% | Low |  |
| Morse LR et al. (2025) | NCT04379011 | double-blind, randomized, placebo-controlled | 12 weeks | 11 | 9 | 0% | High risk |  |
| Pandya R et al. (2024) |  | single-blind, randomized trial | 3 months | 50 | 50 (LEV) | Most common | High risk |  |
| Ryvlin P et al. (2014) | NCT00490035 | Phase 3, double-blind, randomized, placebo-controlled | 12 weeks | 298 | 100 | 5% | Low |  |
| Sachdeva D et al. (2025) | - | randomized, active-controlled | 12 months | 148 | 144 (OXA) | 0% | Some concerns |  |
| Van Paesschen W et al. (2013) | NCT00175929 | Phase 2b, double-blind, randomized, placebo-controlled | 10 weeks | 105 | 52 | 5% | Low |  |
|  | NCT00175825 | Phase 2, double-blind, randomized, placebo-controlled | 7 weeks | 154 | 54 | 5% | Low |  |
|  | NCT00160667 | Phase 2, double-blind, randomized, placebo-controlled | 7 weeks | 102 | 50 | 5% | Low |  |

## **Fig. S1 Overview of results of Risk of Bias rating – LEV**

|  | Low risk | Some concerns | High risk |
| --- | --- | --- | --- |
| Overall risk of bias | 15 | 26 | 42 |
| Bias in selection of the reported result | 82 | 0 | 1 |
| Bias in measurement of the outcome | 37 | 46 | 0 |
| Bias due to missing outcome data | 70 | 0 | 13 |
| Bias due to deviations from intended interventions | 64 | 13 | 6 |
| Bias arising from the randomization process | 25 | 24 | 34 |

## **Fig. S2 Overview of results of Risk of Bias rating – BRV**

|  | Low risk | Some concerns | High risk |
| --- | --- | --- | --- |
| Overall risk of bias | 8 | 5 | 1 |
| Bias in selection of the reported result | 14 | 0 | 0 |
| Bias in measurement of the outcome | 11 | 3 | 0 |
| Bias due to missing outcome data | 14 | 0 | 0 |
| Bias due to deviations from intended interventions | 11 | 3 | 0 |
| Bias arising from the randomization process | 8 | 5 | 1 |

## **Evaluation of AE profiles of LEV and BRV**

### **Table S5 Frequencies of AEs reported in at least 5 studies - LEV**

| **Blood and lymphatic system disorders** | | | | |  |  |
| --- | --- | --- | --- | --- | --- | --- |
| Symptom | All doses | 1000 mg | | 2000 mg | 3000 mg | PLC |
| Anemia | 7: 23/889 2.59% | 1:1/122 0.82% | | 1: 4/204 1.96% | - | 3: 8/258 3.10% |
| Thrombocytopenia | 11: 40/1265 3.16% | 3: 0/324 0.00% | | 1: 1/204 0.49% | 2: 20/131 15.27% | 6: 27/455 5.93%% |
| White blood cell count increased | 5: 6/985 0.61% | 3: 2/410 0.49% | | 1: 0/70 0.00% | 1: 1/70 1.43% | 2: 4/154 2.60% |
|  |  |  | |  |  |  |
| **Cardiac disorders** | | | | |  |  |
| Symptom | All doses | 1000 mg | | 2000 mg | 3000 mg | PLC |
| Myocardial infarction | 5: 5/700 0.71% | 1: 1/122 0.82% | | 1: 1/136 0.74% | - | 2: 0/167 0.00% |
| Palpitation | 9: 10/1340 0.75% | 3: 4/513 0.78% | | 2: 4/340 1.18% | 1: 0/10 0.00% | 2: 2/167 1.20% |
|  |  |  | |  |  |  |
| **Ear and labyrinth disorders** | | | | |  |  |
| Symptom | All doses | 1000 mg | | 2000 mg | 3000 mg | PLC |
| Ear pain | 5: 3/782 0.38% | 1: 0/122 0.00% | | 2: 2/340 0.59% | - | - |
| Vertigo | 12: 113/2185 5.17% | 3: 6/235 2.55% | | 2: 35/340 10.29% | 2: 4/70 5.71% | 3: 3/227 1.32% |
|  |  |  | |  |  |  |
| **Eye disorder** | | | | |  |  |
| Symptom | All doses | 1000 mg | | 2000 mg | 3000 mg | PLC |
| Blurred vision | 9: 10/1280 0.78% | 3: 3/513 0.58% | | 2: 3/340 0.88% | 2: 1/44 2.27% | 3: 4/132 1.15% |
| Diplopia | 10: 8/1555 0.51% | 3: 2/710 0.28% | | 3: 1/387 0.26% | 1: 1/37 2.73% | 3: 2/168 1.19% |
| Visual impairment | 5: 6/844 0.71% | 3: 5/504 0.99% | | 2: 1/340 0.29% | - | - |
|  |  |  | |  |  |  |
| **Gastrointestinal disorder** | | | | |  |  |
| Symptom | All doses | 1000 mg | | 2000 mg | 3000 mg | PLC |
| Abdominal discomfort | 8: 9/1057 0.85% | 3: 5/513 0.97% | | 2: 2/340 0.59% | 2: 2/47 4.26% | 3: 7/204 3.43% |
| **Abdominal pain** | **16: 57/2627 2.17%** | **8: 25/1097 2.28%** | | **4: 7/516 1.36%** | **3: 3/181 1.66%** | **5: 16/444 3.64%** |
| **Constipation** | **20: 40/2059 1.94%** | **7: 14/868 1.61%** | | **3: 4/361 1.11%** | **4: 6/106 5.66%** | **8: 11/324 3.40%** |
| **Diarrhea** | **26: 178/4020 4.43%** | **10: 38/1205 3.15%** | | **4: 14/431 3.25%** | **4: 11/218 5.05%** | **16: 56/1005 5.57%** |
| Dry mouth | 13: 15/1423 1.05% | 2: 1/452 0.22% | | 2: 1/340 0.29% | 2: 1/71 1.41% | 7: 14/343 4.08% |
| Dyspepsia | 18: 60/2618 2.29% | 4: 6/883 0.68% | | 2: 7/340 2.06% | - | 8: 17/604 2.81% |
| Flatulence | 6: 6/1084 0.55% | 2: 5/380 1.32% | | 2: 1/340 0.29% | - | 1: 1/84 1.19% |
| Gastritis | 6: 12/1018 1.18% | 3: 4/441 0.91% | | 1: 2/204 0.98% | 1: 0/10 0.00% | 1: 0/83  0.00% |
| Gastrointestinal discomfort | 5: 4/193 2.21% | - | | 1: 0/21 0.00% | 1: 3/25 12.0% | 4: 8/165 4.85% |
| **Nausea** | **38: 238/4645 5.12%** | **9: 53/1142 4.64%** | | **6: 27/511 5.28%** | **4: 10/93 10.75%** | **21: 58/1047 5.54%** |
| Toothache | 6: 21/897 2.34% | 2: 3/183 1.64% | | 2: 9/340 2.65% | 1: 0/10 0.00% | 1: 0/84 0.00% |
| Upper abdominal pain | 11: 34/1594 2.13% | 3: 5/771 0.49% | | 2: 3/340 0.88% | 1: 0/10 0.00% | 2: 4/118 3.39% |
| Vomiting | 17: 66/2109 3.13% | 5: 17/944 1.80% | | 3: 5/342 1.46% | 1: 0/10 0.00% | 6: 29/365 7.95% |
|  |  |  | |  |  |  |
| **General disorder** | | | | |  |  |
| Symptom | All doses | 1000 mg | | 2000 mg | 3000 mg | PLC |
| Asthenia | 16: 137/2151 6.37% | 6: 33/789 4.18% | | 4: 40/488 8.20% | 2: 38/282 13.48% | 7: 36/548 6.57% |
| Fatigue | 29: 401/3268 12.27% | 8: 104/1029 10.11% | | 2: 20/157 12.73% | 1: 3/60 5.00% | 12: 65/647 10.05% |
| Feeling abnormal | 5: 5/1077  0.46% | 2: 3/452  0.66% | | 2: 1/340  0.29% | - | - |
| Gait disturbance | 7: 14/1356 1.03% | 3: 11/710 1.55% | | 1: 0/204 0.00% | - | 2: 1/167 0.06% |
| Hyperhidrosis | 8: 9/1179  0.76% | 2: 1/452  0.22% | | 2: 2/340  0.59% | 2: 0/62  0.00% | 3: 6/97  6.19% |
| Irritability | 31: 156/2498 6.24% | 6: 42/813 5.17% | | 4: 25/363 6.89% | 2: 1/35 2.86% | 14: 21/655 3.20% |
| Malaise | 6: 19/898  2.12% | 3: 14/513  2.73% | | 2: 4/340  1.18% | 1: 0/10  0.00% | - |
| Oedema peripheral | 6: 11/850  1.29% | 1: 3/122  2.46% | | 2: 4/340  1.18% | - | 1: 2/83  2.41% |
| Pain | 12: 69/1491 4.63% | 4: 21/349 6.02% | | 2: 5/242 2.02% | 2: 17/135 12.59% | 9: 44/623 7.06% |
| Procedural pain | 6: 2/771  0.26% | 2: 0/183  0.00% | | 1: 0/136  0.00% | 1: 1/10  10.00% | 2: 2/167  1.20% |
| Pyrexia | 10: 33/1452 2.27% | 3: 6/513 1.17% | | 2: 5/340 1.47% | 1: 1/10 10.00% | 3: 9/243 3.70% |
| Sweating | 8: 9/1179 0.76% | 2: 1/452 0.22% | | 2: 2/340 0.58% | 2: 0/62 0.00% | 3: 6/97 6.19% |
| Tiredness | 5: 104/339 30.68% | - | | 1: 67/204 32.84% | 2: 24/62 38.71% | 4: 7/129 5.42% |
|  |  |  | |  |  |  |
| **Hepatobiliary disorders** | | | | |  |  |
| Symptom | All doses | 1000 mg | | 2000 mg | 3000 mg | PLC |
| Gamma-Glutamyltranferase increased | 8: 13/978 1.33% | 4: 8/440 1.82% | | 1: 2/204 0.98% | 1: 1/10 10.00% | 1: 1/84 1.19% |
|  |  |  | |  |  |  |
| **Immune system disorders** | | | | |  |  |
| Symptom | All doses | 1000 mg | | 2000 mg | 3000 mg | PLC |
| Face swelling | 5: 5/1050 0.48% | 3: 3/629 0.48% | | 1: 1/136 0.74% | - | - |
| Seasonal allergy | 5: 5/826  0.61% | 1: 0/122  0.00% | | 2: 1/340  0.29% | - | 1: 0/84  0.00% |
|  |  |  | |  |  |  |
| **Infections and infestations** | | | | |  |  |
| Symptom | All doses | 1000 mg | | 2000 mg | 3000 mg | PLC |
| Bronchitis | 6: 7/861 0.81% | 1: 0/122 0.00% | | 2: 5/340 1.47% | - | 1: 1/84 1.19% |
| Cystitis | 7: 13/975  13.33% | 2: 5/183  2.73% | | 2: 1/340  0.29% | 1: 0/10  0.00% | 2: 0/167  0.00% |
| Gastroenteritis | 6: 17/796  2.14% | 2: 4/183  2.19% | | 1: 5/204  2.45% | 1: 1/10  10.00% | 1: 2/84  2.38% |
| Herpes zoster | 5: 5/825  0.61% | 1: 1/122  0.82% | | 2: 1/340  0.29% | - | 1: 2/83  2.41% |
| **Infection** | **9: 95/1349 7.04%** | **3: 37/326 11.35%** | | **3: 9/352 2.56%** | **3: 41/298 13.42%** | **4: 26/351 7.41%** |
| **Influenza** | **15: 88/1884 4.67%** | **4: 14/387 3.62%** | | **3: 10/446 2.24%** | **3: 13/123 10.57%** | **9: 40/733 5.46%** |
| **Nasopharyngitis** | **22: 406/3631 11.18%** | **5: 134/644 20.81%** | | **4: 55/457 12.04%** | **3: 21/140 15.00%** | **10: 60/669 8.97%** |
| Pharyngitis | 15: 66/1847 3.57% | 4: 13/307 4.23% | | 3: 7/446 1.57% | 2: 4/70 5.71% | 9: 26/644 4.04% |
| Pneumonia | 9: 10/1011  0.99% | 3: 7/383  1.83% | | 1: 1/136  0.74% | - | 5: 8/326 |
| Sinusitis | 6: 13/847  1.53% | 1: 0/122  0.00% | | 3: 8/361  2.21% | - | 2: 0/108  0.00% |
| Upper respiratory tract infection | 12: 110/1426 7.71% | 4: 19/434 4.38% | | 2: 41/340 12.06% | - | 4: 17/267 6.44% |
| Urinary tract infection | 13: 58/1833 3.16% | 4: 20/469 4.26% | | 4: 6/488 1.23% | - | 8: 27/521 5.18% |
| Viral infection | 5: 7/657  1.07% | 1: 0/122  0.00% | | 1: 1/136  0.74% | - | 1: 3/84  3.57% |
|  |  |  | |  |  |  |
| **Injury, poisoning and procedural complications** | | | | |  |  |
| Symptom | All doses | 1000 mg | | 2000 mg | 3000 mg | PLC |
| Accidental injury | 6: 83/1058 7.84% | 3: 29/489 5.93% | | 2: 15/148 10.14% | 2: 17/282 6.03% | 5: 66/448 14.73% |
| Arthropod sting | 7: 6/975  0.62% | 2: 0/183  0.00% | | 2: 1/340  0.29% | 1: 1/10  10.00% | 2: 0/167  0.00% |
| Fall | 10: 21/984 2.13% | 3: 10/206 4.85% | | 2: 2/340 0.59% | 2: 1/35 2.86% | 3: 12/133  9.02% |
| Head injury | 9: 12/1077 1.11% | 3: 2/216 0.93% | | 2: 2/340 0.59% | 1: 2/10 20.00% | 2: 3/167  1.80% |
| Thermal burn | 7: 10/901 1.11% | 3: 7/356 1.97% | | 1: 1/136 0.74% | 1: 0/10 0.00% | 1: 1/84 1.19% |
| Tooth fracture | 5: 5/613  0.82% | - | | 1: 2/136  1.47% | - | 2: 3/167  1.80% |
|  |  |  | |  |  |  |
| **Investigations** | | | | |  |  |
| Symptom | All doses | 1000 mg | | 2000 mg | 3000 mg | PLC |
| Blood cholesterol increased | 5: 8/782  1.02% | 2: 6/340  1.76% | | - | - | 2: 0/167  0.00% |
| Weight gain | 15: 103/2797 3.68% | 4: 21/771 2.72% | | 2: 3/340 0.88% | 1: 2/10 20.00% | 2: 3/94 3.19% |
| Weight loss | 18: 64/2771 2.31% | 5: 14/786 1.78% | | 3: 5/361 1.39% | 1: 0/10 0.00% | 4: 3/141 2.13% |
|  |  |  | |  |  |  |
| **Metabolism and nutrition disorders** | | | | |  |  |
| Symptom | All doses | 1000 mg | | 2000 mg | 3000 mg | PLC |
| Anorexia | 11: 41/1031 3.98% | - | | 3: 6/361 1.66% | - | 4: 9/215 4.19% |
| Decreased appetite | 17: 39/2052 1.90% | 4: 12/771 1.56% | | 2: 2/340 0.59% | 1: 0/10 0.00% | 5: 10/292 3.42% |
| Increased appetite | 5: 4/910  0.44% | 3: 4/710  0.56% | | 1: 0/136  0.00% | - | 1: 3/34  8.82% |
|  |  |  | |  |  |  |
| **Musculoskeletal and connective tissue disorders** | | | | |  |  |
| Symptom | All doses | 1000 mg | | 2000 mg | 3000 mg | PLC |
| Arthralgia | 10: 32/1406 2.28% | 3: 10/513 1.95% | | 2: 4/340 118% | 2: 3/47 6.38% | 4: 21/270 7.78% |
| Back pain | 15: 77/2020 3.81% | 5: 22/472 4.66% | | 3: 13/446 2.91% | 1: 0/10 0.00% | 8: 37/712 5.20% |
| Contusion | 10: 27/1621 1.67% | 4: 7/583 1.20% | | 3: 7/410 1.71% | 2: 3/80 3.75% | 3: 9/237 3.80% |
| Ligament sprain | 5: 6/554  1.08% | 2: 4/183  2.19% | | 1: 0/204  0.00% | 1: 1/10  10.00% | 2: 3/167  1.80% |
| Muscle spasms | 6: 11/904  1.22% | 1: 5/122  4.10% | | 2: 4/340  1.18% | - | 2: 1/167  0.60% |
| Musculoskeletal pain | 6: 15/690  2.17% | 2: 4/183 2.19% | | 2: 9/340  2.65% | 1: 1/10  10.00% | 2: 1/167  0.60% |
| Musculoskeletal stiffness | 5: 5/818  0.61% | 2: 3/183  1.64% | | 2: 1/340  0.29% | 1: 0/10  0.00% | - |
| Myalgia | 11: 18/1370 1.31% | 3: 4/470 0.85% | | 2: 3/340 0.88% | - | 5: 6/267 2.25% |
| Neck pain | 9: 23/102 2.09% | 1: 2/122 1.64% | | 2: 3/340 0.88% | 2: 6/85 7.06% | 5: 4/380 1.05% |
| Pain in extremity | 11: 27/1397 1.93% | 4: 11/523 2.10% | | 2: 3/340 0.88% | 1: 0/10 0.00% | 3: 5/233 2.15% |
|  |  |  | |  |  |  |
| **Nervous system disorders** | | | | |  |  |
| Symptom | All doses | 1000 mg | | 2000 mg | 3000 mg | PLC |
| Agitation | 16: 42/1676 2.51% | 5: 17/794 2.14% | | 2: 5/340 1.47% | 3: 3/98 3.06% | 9: 10/532 1.88% |
| Aphasia | 7: 6/1069 0.56% | 4: 4/751 0.53% | | 1: 1/136 0.74% | - | - |
| Ataxia | 9: 10/1313 0.76% | 4: 3/762 0.39% | | 2: 3/206 1.46% | - | 2: 0/26 0.00% |
| Balance disorders | 7: 16/999 1.60% | 2: 5/452 1.11% | | 1: 3/136 2.21% | - | 2: 2/86 2.33% |
| Concentration problems | 12: 36/1574 2.29% | 4: 8/743 1.08% | | 3: 9/361 2.49% | - | 3: 3/110 2.73% |
| Confusion | 10: 14/1356 1.03% | 3: 9/493 1.83% | | 3: 1/361 0.28% | 1: 0/60  0.00% | 4: 1/251 0.40% |
| Convulsion | 12: 87/2243 3.88% | 6: 21/1028 2.04% | | 2: 13/242 5.37% | - | 4: 24/351 6.84% |
| Disorientation | 5: 3/741  0.40% | 2: 1/380  0.26% | | 3: 2/361  0.55% | - | 1: 0/24  0.00% |
| **Dizziness** | **57: 554/5754 9.63%** | **14: 159/1545 10.29%** | | **7: 42/424 9.90%** | **12: 67/750 8.93%** | **34: 109/1793 6.08%** |
| Drowsiness | 6: 19/159 11.95% | 2: 6/67 8.96% | | - | - | 2: 2/17 11.76% |
| Dysarthria | 8: 8/1171 0.68% | 4: 3/720 0.42% | | 2: 3/340 0.88% | - | 2: 1/100 1.00% |
| Dysgeusia | 5: 5/1077  0.46% | 2: 2/452  0.44% | | 2: 4/340  1.18% | - | - |
| Epilepsy | 5: 36/712  5.06% | 2: 23/145  15.86% | | 1:11/204  5.39% | - | 2: 3/108  2.78% |
| **Headache** | **52: 794/5729 13.86%** | **15: 167/1600 10.43%** | | **6: 111/584 19.00%** | **9: 53/621 8.53%** | **28: 227/1535 14.79%** |
| Hypoaesthesia | 8: 8/1262 0.63% | 3: 3/513 0.58% | | 2: 2/340 0.59% | 1: 0/10 0.00% | 1: 0/84 0.00% |
| Impaired coordination | 6: 12/589 2.04% | 2: 0/380 0.00% | | 1: 1/136 0.74% | 1: 5/34 14.71% | 3: 1/68 1.47% |
| Lethargy | 10: 17/1536 1.11% | 4: 9/720 1.25% | | 2: 2/340 0.59% | 1: 3/89 3.37% | 2: 0/172 0.00% |
| Memory impairment | 13: 39/1792 2.18% | 4: 21/743 2.83% | | 4: 8/408 1.96% | - | 3: 6/155 3.87% |
| Migraine | 9: 16/1665 0.97% | 4: 8/883 0.91% | | 2: 4/340 1.18% | - | 2: 0/167 0.00% |
| Paraesthesia | 12: 22/1495 1.47% | 3: 4/467 0.86% | | 3: 4/361 1.11% | 2: 2/62 3.23% | 5: 17/236 7.20% |
| Partial seizures | 5: 4/724  0.55% | 1: 2/122  1.64% | | 1: 0/204  0.00% | - | 1: 1/83  1.20% |
| Personality change | 5: 12/1015  1.18% | 3: 3/710  0.42% | | 1: 1/204  0.49% | - | 1: 7/97  7.22% |
| Psychomotor hyperactivity | 6: 11/880 1.25% | 2: 3/588 0.51% | | 1: 0/136 0.00% | - | 1: 5/34 14.71% |
| Restlessness | 5: 10/933  1.07% | 2: 1/380  0.26% | | 1: 2/204  0.98% | - | 1: 4/66  6.06% |
| Sedation | 10: 39/1145 3.41% | 3: 9/603 1.49% | | 2: 3/157 1.91% | - | 4: 20/103 19.42% |
| **Somnolence** | **47: 733/5768 12.71%** | **12: 132/1407 9.38%** | | **7: 80/607 13.18%** | **9: 85/599 14.19%** | **22: 125/1397 8.95%** |
| Tremor | 16: 76/2735 2.78% | 3: 11/710 1.55% | | 2: 11/340 3.24% | - | 5: 12/251 4.78% |
|  |  |  | |  |  |  |
| **Psychiatric disorders** | | | | |  |  |
| Symptom | All doses | 1000 mg | | 2000 mg | 3000 mg | PLC |
| Abnormal behaviour | 9: 39/1161 3.36% | 2: 22/588 3.94% | | - | - | 2: 1/118 0.85% |
| Abnormal dreams | 7: 3/1073 0.28% | 2: 1/588 0.17% | | 1: 0/136 0.00% | - | 3: 3/50 6.00% |
| Affect Lability | 6: 12/1314 0.91% | 2: 3/588 0.51% | | 2: 1/340 0.29% | - | 1: 4/97 4.12% |
| Aggression | 11: 65/1558 4.17% | 3: 27/710 3.80% | | 2: 18/340 5.29% | 1: 2/60  3.33% | 4: 4/188 2.13% |
| Altered mood | 7: 37/896 4.13% | 4: 21/641 3.28% | | - | - | 2: 2/54 3.74% |
| Amnesia | 6: 6/1067 0.56% | 2: 4/588 0.68% | | 2: 1/340 0.29% | 1: 1/60  1.67% | 2: 3/144 2.08% |
| Anger | 6: 21/1152 1.82% | 3: 12/873 1.37% | | 1: 3/136 2.21% | - | 2: 2/150 1.33% |
| Anxiety | 21: 64/1962 3.26% | 4: 16/733 2.18% | | 4: 6/363 1.65% | - | 10: 26/498 5.22% |
| Apathy | 8: 12/1439 0.83% | 3: 5/710 0.70% | | 1: 6/204 2.94% | 1: 0/60  0.00% | 3: 2/241 0.83% |
| Cognitive disorder | 9: 3/1131 0.22% | 3: 2/710 0.28% | | 3: 0/342 0.00% | - | 2: 2/17 11.76% |
| Depressed mood | 14: 67/1780 3.76% | 4: 35/883 3.96% | | 2: 9/340 265% | 1: 0/60  0.00% | 6: 18/266 6.77% |
| **Depression** | **19: 155/3300 4.70%** | **7: 36/998 3.61%** | | **4: 25/467 5.35%** | **4: 12/352 3.41%** | **10: 20/724 2.76%** |
| Hallucination | 5: 4/654  0.61% | 3: 2/475  0.42% | | - | - | 3: 3/205  1.46% |
| **Insomnia** | **27: 120/3057 3.93%** | **8: 48/1138 4.22%** | | **5: 17/509 3.34%** | **4: 29/354 8.19%** | **14: 40/833 4.80%** |
| Libido loss | 6: 9/1123 0.80% | 2: 0/588 0.00% | | 2: 2/225 0.89% | - | 1: 1/24 4.17% |
| Mental change | 5: 8/520 1.54% | 2: 3/163 1.84% | | - | 1: 3/37 8.11% | 1: 0/37 0.00% |
| **Mood swings** | **10: 20/1429 1.40%** | **2: 9/588 1.53%** | | **2: 4/340 1.18%** | **2: 1/97 1.03%** | **3: 3/181 1.66%** |
| **Nervousness** | **13: 59/1867 3.16%** | **4: 14/499 2.81%** | | **4: 11/488 2.25%** | **3:13/342 3.80%** | **7: 13/592 2.20%** |
| Panic attack | 6: 6/1335 0.45% | 3: 2/710 0.28% | | 2: 4/340 1.18% | - | - |
| Psychotic disorder | 5: 3/320  0.94% | 2: 0/155  0.00% | | - | - | 1: 0/97  0.00% |
| Sleep disorder | 8: 32/1373 2.33% | 3: 5/710 0.70% | | 1: 22/204 10.78% | 1: 2/60  3.33% | 2: 1/144 0.69% |
| Suicidal ideation | 8: 9/1237 0.73% | 3: 4/710 0.56% | | 2: 3/340 0.88% | - | 2: 0/167 0.00% |
| Dysmenorrhoea | 5: 14/775  1.81% | 1: 5/61  8.20% | | 2: 0/340  0.00% | 1: 0/10  0.00% | 1: 3/84  3.57% |
| Erectile dysfunction | 6: 4/735  0.54% | 1: 1/122  0.82% | | 2: 1/225  0.44% | - | 2: 1/107  0.93% |
|  |  |  | |  |  |  |
| **Respiratory, thoracic and mediastinal disorders** | | | | |  |  |
| Symptom | All doses | 1000 mg | | 2000 mg | 3000 mg | PLC |
| Cough | 11: 42/1097 3.83% | 2: 12/140 8.57% | | 2: 11/340 3.24% | 1: 2/12 16.67% | 6: 11/293 3.75% |
| Dyspnoea | 5: 6/826  0.73% | 1: 2/122  1.64% | | 2: 1/340  0.29% | - | 1: 1/84  1.19% |
| Epistaxis | 11: 17/1603 1.06% | 4: 5/771 0.65% | | 2: 5/340 1.47% | 1: 0/10 0.00% | 3: 4/134 2.99% |
| Oropharyngeal pain | 5: 13/376  3.46% | 2: 7/183  3.83% | | 1: 1/136  0.74% | 2: 2/22  9.09% | 1: 3/8  37.50% |
| Nasal congestion | 7: 18/731 2.46% | - | | 1: 3/136 2.21% | - | 5: 4/292 1.37% |
| Rhinorrhoea | 5: 10/619  1.62% | 2: 6/234  2.56% | | 2: 4/340  1.18% | 1: 0/10  0.00% | - |
| **Rhinitis** | **12: 56/2060 2.72%** | **5: 24/405 5.93%** | | **3: 2/352 0.57%** | **3: 10/292 3.42%** | **7: 22/550 4.00%** |
|  |  |  | |  |  |  |
| **Skin and subcutaneous tissue** | | | | |  |  |
| Symptom | All doses | 1000 mg | 2000 mg | | 3000 mg | PLC |
| Acne | 8: 6/1478 0.41% | 3: 1/710 0.14% | 2: 2/340 0.59% | | - | 2: 3/118 2.54% |
| Alopecia | 8: 23/2201 1.04% | 3: 6/710 0.85% | 2: 2/340 0.59% | | - | - |
| Dermatitis allergic | 6: 4/1234 0.32% | 3: 2/710 0.28% | 1: 1/204 0.49% | | - | - |
| Dry skin | 6: 9/1148  0.78% | 3: 4/513  0.78% | 2: 3/340  0.88% | | 1: 1/10  10.00% | - |
| Eczema | 6: 9/1047  0.86% | 3: 2/513  0.39% | 1: 1/204  0.49% | | 1: 1/10  10.00% | - |
| **Pruritus** | **16: 43/1659 2.59%** | **4: 14/686 2.04%** | **4: 9/408 2.21%** | | **3: 2/69 2.89%** | **5: 4/161 2.48%** |
| **Rash** | **27: 95/3732 2.55%** | **5: 17/944 1.80%** | **3: 18/361 4.99%** | | **3: 3/72 4.17%** | **12: 23/667 3.45%** |
| Skin laceration | 5: 4/642  0.62% | - | 1: 1/136  0.74% | | - | 3: 9/233  3.86% |
|  |  |  |  | |  |  |
| **Vascular disorders** | | | | |  |  |
| Symptom | All doses | 1000 mg | | 2000 mg | 3000 mg | PLC |
| Flushing | 6: 3/861  0.35% | 1: 0/122  0.00% | | 2: 2/340  0.59% | - | 1: 1/84  1.19% |
| Hypertension | 9: 18/1212 1.49% | 3: 4/356 1.12% | | 2: 5/340 1.47% | 1: 0/10 0.00% | 3: 10/233 4.29% |
| Syncope | 7: 16/956  1.67% | 2: 3/174  1.72% | | 2: 5/340  1.47% | - | 2: 2/167  1.20% |

### **Table S6 Frequencies of AEs reported in at least 5 studies – BRV**

| **Blood and lymphatic system disorders** | | | |  |  |  |
| --- | --- | --- | --- | --- | --- | --- |
| Symptom | All doses | 50 mg | 100 mg | 150 mg | 200 mg | PLC |
| Erythropenia | 6: 1/1717  0.06% | 5: 0/360  0.00% | 3: 0/440  0.00% | 2: 1/221  0.54% | 1: 0/250  0.00% | 6: 1/686  0.15% |
| Leukopenia | 6: 3/1717  0.17% | 5: 0/360  0.00% | 3: 2/440  0.45% | 2: 1/221  0.54% | 1: 0/250  0.00% | 6: 2/686  0.29% |
| Lymphocyte count decreased | 6: 1/1717  0.06% | 5: 0/360  0.00% | 3: 1/440  0.23% | 2: 0/221  0.00% | 1: 0/250  0.00% | 6: 1/686  0.15% |
| Lymphopenia | 6: 1/1717  0.06% | 5: 0/360  0.00% | 3: 1/440  0.23% | 2: 0/221  0.00% | 1: 0/250  000% | 6: 1/686  0.15% |
| Neutropenia | 6: 13/1717  0.76% | 5: 3/360  0.83% | 3: 4/440  0.68% | 2: 2/221  0.90% | 1: 0/250  0.00% | 6: 1/686  0.15% |
| Neutrophil count decreased | 6: 4/1717  0.23% | 5: 0/360  0.00% | 3: 0/440  0.00% | 2: 1/221  0.54% | 1: 1/250  0.40% | 6: 2/686  0.29% |
| Platelet count decreased | 6: 4/1717  0.23% | 5: 0/360  0.00% | 3: 4/440  0.91% | 2: 2/221  0.90% | 1: 0/250  0.00% | 6: 0/686  0.00% |
| Red blood cell count decreased | 6: 2/1717  0.12% | 5: 1/360  0.28% | 3: 1/440  0.23% | 2: 0/221  0.00% | 1: 0/250  0.00% | 6: 0/686  0.00% |
| Thrombocytopenia | 7: 2/1755  0.11% | 5: 0/360  0.00% | 3: 1/440  0.23% | 3: 1/239  0.42% | 1: 0/250  0.00% | 7: 1/704  0.14% |
| White blood cell count decreased | 6: 0/1717  0.00% | 5: 0/360  0.00% | 3: 0/440  0.00% | 2: 0/221  0.00% | 1: 0/250  0.00% | 6: 2/686  0.29% |
|  |  |  |  |  |  |  |
| **Ear and labyrinth disorders** | | | |  |  |  |
| Symptom | All doses | 50 mg | 100 mg | 150 mg | 200 mg | PLC |
| Vertigo | 5: 35/1263  2.77% | 3: 3/247  1.21% | 3: 13/368  3.53% | - | 2: 13/301  4.32% | 4: 11/509  2.16% |
|  | | | |  |  |  |
| **Gastrointestinal disorders** | | | |  |  |  |
| Symptom | All doses | 50 mg | 100 mg | 150 mg | 200 mg | PLC |
| Abdominal pain | 5: 13/1180  1.10% | 3: 5/216  2.31% | 2: 3/353  0.85% | 2: 1/36  2.78% | 1: 1/250  0.40% | 5: 9/493  1.83% |
| Abdominal pain upper | 5: 20/1171  1.71% | 2: 6/216  2.78% | 2: 4/353  1.13% | 2: 0/36  0.00% | 1: 3/250  1.20% | 5: 7/493  1.41% |
| Constipation | 6: 24/1182  2.03% | 3: 6/216  2.78% | 2: 4/353  1.13% | 2: 1/36  2.78% | 2: 7/261  2.68% | 6: 3/502  5.98% |
| Diarrhea | 6: 33/1182  2.79% | 3: 7/216  3.24% | 2: 6/353  1.70% | 2: 1/36  2.78% | 2: 9/261  3.45% | 6: 14/502  2.79% |
| Nausea | 11: 93/2035 4.57% | 6: 16/384 4.17% | 4: 18/388 4.64% | 3: 5/88 5.68% | 2: 5/301 1.66% | 8: 29/716  4.05% |
| Vomiting | 5: 29/1266  2.29% | 4: 12/300  4.00% | 3: 5/368  1.36% | 1: 3/52  5.77% | 1: 3/250  1.20% | 4: 5/511  9.78% |
|  |  |  |  |  |  |  |
| **General disorders** | | |  |  |  |  |
| Symptom | All doses | 50 mg | 100 mg | 150 mg | 200 mg | PLC |
| Asthenia | 5: 17/1263  1.35% | 3: 8/247  3.24% | 3: 6/368  1.63% | - | 2: 5/301  1.66% | 4: 7/509  1.38% |
| **Fatigue** | **12: 165/2052 8.04%** | **8: 37/491 7.54%** | **5: 38/475**  **8.00%** | **4: 18/257 7.00%** | **3: 37/312 11.86%** | **10: 31/779 3.98%** |
| Irritability | 8: 45/1790 2.51% | 6: 19/407 4.66% | 4: 11/455 2.42% | 2: 2/221 0.90% | 2: 7/261 2.68% | 7: 11/695 1.58% |
| Pyrexia | 5: 9/1171  0.77% | 3: 1/216  4.63% | 2: 1/353  0.28% | 2: 0/36  0.00% | 1: 0/250  0.00% | 5: 4/493  0.81% |
|  |  |  |  |  |  |  |
| **Infections and infestations** | | |  |  |  |  |
| Symptom | All doses | 50 mg | 100 mg | 150 mg | 200 mg | PLC |
| Nasopharyngitis | 10: 91/2084 4.37% | 7: 23/535 4.30% | 4: 14/388 3.61% | 3: 5/88  3.41% | 2: 19/398 4.77% | 8: 38/815 4.66% |
| Urinary tract infection | 9: 54/1725 3.13% | 7: 17/535 3.18% | 4: 15/388 3.86% | 3: 2/88 2.27% | 2: 11/398 2.76% | 7: 22/694 3.17% |
|  |  |  |  |  |  |  |
| **Injury poisoning and procedural complications** | | | |  |  |  |
| Symptom | All doses | 50 mg | 100 mg | 150 mg | 200 mg | PLC |
| Fall | 5: 13/1171  1.11% | 3: 0/216  0.00% | 2: 4/353  1.13% | 2: 0/36  0.00% | 1: 3/250  1.20% | 5: 10/493  2.03% |
| Laceration | 6: 15/1717  0.82% | 5: 1/360  0.27% | 3: 3/440  0.68% | 2: 5/221  2.26% | 1: 2/250  0.80% | 6: 3/686  0.44% |
|  |  |  |  |  |  |  |
| **Metabolic and nutrition disorders** | | | |  |  |  |
| Symptom | All doses | 50 mg | 100 mg | 150 mg | 200 mg | PLC |
| Decreased appetite | 5: 25/1249  2.00% | 4: 13/315  4.13% | 4: 5/388  1.29% | - | 1: 4/250  1.60% | 3: 4/459  0.87% |
| Weight decreased | 5: 24/1436  1.67% | 3: 8/351  2.23% | 2: 3/353  0.85% | 1: 1/18  5.00% | 2: 7/398  1.76% | 5: 3/626  0.48% |
| Weight increased | 5: 11/1285  0.86% | 2: 3/200  1.50% | 2: 1/353  0.28% | 1: 0/18  0.00% | 1: 1/250  0.40% | 4: 4/477  0.84% |
|  |  |  |  |  |  |  |
| **Musculoskeletal and connective tissue disorders** | | | | | | |
| Symptom | All doses | 50 mg | 100 mg | 150 mg | 200 mg | PLC |
| Back pain | 6: 31/1601  1.94% | 3: 8/253  3.16% | 2: 4/353  1.13% | 2: 1/70  1.43% | 1: 2/250  0.80% | 6: 17/650  2.62% |
| Pain in extremity | 5: 19/1225  1.55% | 3: 7/268  2.61% | 3: 4/373  1.07% | 1: 1/18  5.56% | 1: 2/250  0.80% | 4: 6/477  1.26% |
|  |  |  |  |  |  |  |
| **Nervous system disorders** | | |  |  |  |  |
| Symptom | All doses | 50 mg | 100 mg | 150 mg | 200 mg | PLC |
| Balance disorder | 5: 12/1171  1.02% | 3: 3/216  1.39% | 2: 2/353  5.67% | 2: 2/36  5.56% | 1: 3/250  1.20% | 5: 1/493  0.20% |
| Convulsion | 10: 64/1939 3.30% | 8: 16/491 3.26% | 5: 19/475 4.00% | 4: 6/257 2.33% | 1: 3/250  1.20% | 5: 14/261 5.36% |
| **Dizziness** | **13: 231/2351 9.83%** | **9: 62/642 9.66%** | **5: 47/475 9.89%** | **4: 15/257 5.84%** | **4: 64/460 13.91%** | **11: 52/928 5.60%** |
| **Headache** | **15: 249/2549 9.77%** | **9: 79/642 12.31%** | **5: 41/475 8.42%** | **4: 26/257 17.90%** | **4: 32/460 6.97%** | **11: 99/928 10.67%** |
| Memory impairment | 8: 17/1817  0.94% | 6: 5/407  1.23% | 4: 7/455  1.54% | 3: 2/239  0.84% | 1: 3/250  1.20% | 7: 10/704  1.42% |
| **Somnolence** | **12: 349/2426 14.39%** | **8: 62/595 10.42%** | **4: 70/460**  **15.23%** | **4: 20/257 7.78%** | **3: 78/449 17.37%** | **10: 65/919 7.07%** |
| Tremor | 9: 14/1800  0.78% | 6: 4/376  1.06% | 3: 6/440  1.36% | 4: 5/257  1.96% | 2: 4/261  1.53% | 9: 14/729  1.92% |
|  |  |  |  |  |  |  |
| **Psychiatric disorders** | | |  |  |  |  |
| Symptom | All doses | 50 mg | 100 mg | 150 mg | 200 mg | PLC |
| Aggression | 9: 23/1937  1.19% | 6: 3/376  0.80% | 3: 2/440  0.45% | 4: 2/257  0.78% | 1: 2/250  0.80% | 8: 6/720  0.83% |
| Anxiety | 10: 36/1916  1.88% | 7: 15/475  3.16% | 5: 7/475  1.47% | 3: 2/239  0.84% | 2: 9/261  3.45% | 8: 8/713  1.12% |
| Depressed mood | 7: 7/1755  0.40% | 5: 3/360  0.83% | 3: 1/440  0.23% | 3: 2/239  0.84% | 1: 1/250  0.40% | 7: 8/704  1.14% |
| Depression | 9: 42/1905  2.20% | 7: 20/475  4.21% | 5: 10/475  2.11% | 3: 4/239  1.67% | 1: 3/250  1.20% | 7: 9/704  1.28% |
| Insomnia | 8: 34/1426 2.38% | 6: 20/384 5.21% | 4: 7/388 1.80% | 3: 3/88 3.41% | 1: 6/250  2.40% | 6: 11/545 2.02% |
| Nervousness | 6: 12/1717  0.70% | 5: 5/360  1.38% | 3: 3/440  0.68% | 2: 0/221  0.00% | 1: 1/250  0.40% | 6: 3/686  0.43% |

### **Table S7 All reported AEs in less than 5 studies- LEV**

| **System Organ Class** | **Adverse events (frequency)** |
| --- | --- |
| Blood and lymphatic system disorders | Anaemia hypochromic (0), Anaemia vitamin B12 deficiency (1), Bicytopenia (0), Eosinophilia (3), Erythrocytes decreased (0), Erythrocytes increased (1), Granulocytopenia (1), Iron deficiency anaemia (0), Leukocytes decreased (2), Leukocytes increased (1), Leucocytosis (2), Leukopenia (5), Lymphadenitis (0), Lymphadenopathy (2), Lymphocyte count decreased (0), Monocyte count decreased (0), Neutropenia (13), Neutrophilia (1), Neutrophil count increased (4), Normocytic anaemia (0), Pancytopenia (1), White blood cell count decreased (3) |
| Cardiac disorders | Acute coronary syndrome (1), Acute myocardial infarction (1), Angina pectoris (4), Angina unstable (1), Aortic valve incompetence (1), Arrhythmia (0), Atrial fibrillation (2), Atrioventricular block complete (1), Atrioventricular block first degree (1), Atrioventricular block second degree (1), Bradycardia (3), Cardiac aneurysm (1), Cardiac failure (4), Cardiac flutter (1), Cardiovascular disorder (0), Cardiovascular insufficiency (4), Coronary artery disease (2), Coronary artery occlusion (0), Coronary artery stenosis (1), Diastolic dysfunction (0), ECG abnormalities (4), Extrasystoles (1), Heart rate increased (2), Heart surgery (0), Mitral valve incompetence (0), Myocardial infarction (1), Myocardial necrosis marker increased (0), Patent foramen ovale (1), Sinus tachycardia (1), Tachycardia (3), Tricuspid valve incompetence (0), Vaccination site lymphadenopathy (0), Ventricular extrasystoles (1), Ventricular hypertrophy (0), Ventricular hypokinesia (1), Ventricular tachycardia (0) |
| Congential, familial and genetic disorders | Epidermolysis (0), Odontogenic cyst (0), Stevens-Johnson-Syndrome (1) |
| Ear and labyrinth disorders | Cerumen impaction (0), Deafness unilateral (1), Ear disorder (3), Eustachian tube disorder (0), Hearing impairment (1), Hyperacusis (0), Hypoacusis (4), Meniere’s Disease (2), Middle ear effusion (1), Middle ear infection (1), Stye (1), Sudden hearing loss (0), Tinnitus (5), Tympanic membrane perforation (0) |
| Endocrine disorders | Hirsutism (1), Hyperthyroidism (1) |
| Eye disorders | Abnormal sensation in eye (0), Asthenopia (3), Cataract (4), Chalazion (0), Conjunctival haemorrhage (1), Conjunctivitis (3), Conjunctivitis allergic (0), Corneal bleeding (0), Corneal transplant (0), Dry eye (0), Exophthalmos (0), Eye allergy (1), Eye disorder (3), Eye haemorrhage (1), Eye inflammation (1), Eye irritation (1), Eye pain (1), Eye pruritus (0), Eye redness (1), Eyelid oedema (1), Eye ptosis (1), Flashing lights (1), Glaucoma (0), Iridocyclitis (0), Lacrimation increased (1), Myopia (0), Ocular hyperaemia (0), Photopsia (0), Saccadic eye movement (2), Scintillating scotoma (0), Staring (1), Strabismus (0), Vision change (3), Visual acuity reduced (4), Vitreous detachment (1) |
| Gastrointestinal disorders | Altered taste (0), Appendicitis (1), Stomach discomfort (3), Abdominal distension (13), Abdominal fissure (1), Anal incontinence (2), Aphthous stomatitis (1), Bruxism (4), Colitis (0), Colonic polyp (0), Dental caries (9), Dental repair (0), Dental treatment (0), Distended abdomen (3), Diverticulum (2), Diverticulum intestinal (1), Drooling (1), Duodenitis (1), Dysphagia (3), Eructation (0), Faecal occult blood positive (0), Faeces discoloured (1), Faeces hard (0), Feeding tube complications (1), Food poisoning (1), Gastric disorder (1), Gastritis bacterial (1), Gastritis erosive (1), Gastritis haemorrhagic (1), Gastrointestinal disorder (2), Gastrointestinal haemorrhage (0), Gastrointestinal motility disorder (1), Gastrointestinal pain (1), Gastroesophageal reflux disease (6), Gingival bleeding (1), Gingival hypertrophy (1), Gingival pain (1), Gingival ulceration (0), Gum pain (1), Haematochezia (1), Haemorrhoids (0), Hiatus hernia (0), Hiccups (1), Hunger (0), Hypoesthesia oral (1), Ileus (0), Intestinal functional disorder (0), Intestinal ischemia (0), Intestinal polyp (1), Irritable bowel syndrome (2), Lip bleeding (1), Lip pain (0), Lip ulceration (2), Loose stools (1), Lower abdominal pain (1), Melaena (0), Metabolic acidosis (0), Mouth swelling (0), Mouth ulceration (1), Oesophagitis (0), Oral pain (1), Pancreatitis (2), Paraesthesia oral (0), Peptic ulcer (2), Procedural nausea (0), Pulpitis dental (1), Rectal haemorrhage (1), Reflux esophagitis (1), Salivary hypersecretion (1), Small intestinal obstruction (0), Stomatitis (3), Subileus (0), Swollen tongue (1), Thirst (3), Tongue discolouration (0), Tongue disease (0), Tongue ulcer (0), Tooth disorder (0), Tooth grinding (1) |
| General disorders | Alcohol withdrawal syndrome (1), Burning sensation (0), Chest discomfort (1), Chest pain (5), Chills (2), Cold sweat (5), Device failure (1), Device occlusion (0), Discomfort (47), Drug interaction (0), Energy increased (2), Facial pain (2), Feeling cold (5), Feeling drunk (1), Feeling heavy (0), Feeling hot (5), Feeling hot and cold (1), Feeling jittery (1), Feeling sick (1), Feeling warm (1), General physical health deterioration (1), Hernia (0), Hypohidrosis (0), Incisional pain (3), Inflammation localized (1), Influenza-like illness (6), Injection site pain (0), Localized oedema (0), Myofascial pain syndrome (0), Oedema (2), Performance status decreased (1), Physical assault (0), post-traumatic syndrome (1), Restricted mobility (1), Sluggishness (1), Sudden death (2), Swelling (1), Temperature intolerance (1), Vaccination complication (0), Vaccination site pain (1), Vitamin B12 deficiency (1) |
| Hepatobiliary disorders | Aminotransferase elevated (4), Ammonia decreased (1), Choalangitis (1), Cholecystitis (0), Cholelithiasis (1), Cytolytic hepatitis (0), Gallbladder cholesterolosis (0), Hepatic enzymes increased (2), Hepatic steatosis (1), Hepatitis (2), Hepatomegalie (1), Hepatotoxicity (9), Hyperbilirubinaemia (2), Increase in alanine aminotransferase (8), Increase in aspartate aminotransferase (6), Liver disorder (0), Liver function test abnormal (2), Liver injury (2) |
| Immune system disorders | Drug hypersensitivity (2), Idiosyncratic reaction (0), Multiple allergies (0) |
| Infections and infestations | Acute sinusitis (1), Acute tonsillitis (1), Adenoiditis (1), Arthritis infective (1), Bacterial infection (1), Bacteriuria (1), Body tinea (0), Boil (1), Borrelia infection (1), Bronchitis acute (2), Bronchitis bacterial (1), Bronchopneumonia (1), Chlamydial infection (1), Cholecystitis infective (0), COVID-19 (3), Diverticulitis (0), Ear infection (1), Enteritis (1), Epididymal infection (0), Erysipelas (0), Erythema migrans (2), Eye infection (1), Eyelid infection (0), Fungal infection (8), Fungal rash (1), Gastroenteritis enteroviral (2), Gastrointestinal infection (3), Genital infection (0), Gingivitis (6), Helicobacter infection (0), Herpes corneae (1), Herpes simplex (0), HIV test positive (1), Hordeolum (2), Impetigo (1), Infected dermal cyst (1), Infectious peritonitis (0), Infection requires antibiotics (3), Localized infection (0), Mycoplasma infection (0), Nail bed infection (1), Nail infection (0), Onychomycosis (3), Oral herpes (4), Orchitis (0), Otitis externa (3), Otitis media (4), Parotitis (1), Pharyngitis streptococcal (2), Pneumococcal infection (1), Postoperative infection (1), Prostate infection (1), Pyelonephritis (1), Rash pustular (2), Respiratory tract infection viral (5), Rubella (0), Salpingitis (1), Scabies infestation (1), Scarlet fever (1), Sepsis (0), Sinobronchtis (0), Sinus inflammation (2), Sinus node dysfunction (3), Staphylococcal infection (1), Staphylococcal pharyngitis (1), Systemic viral infection (0), Tinea infection (1), Tinea pedis (3), Tinea versicolor (0), Tonsillitis (4), Tooth abscess (4), Tooth infection (6), Tracheitis (1), Tracheobronchitis (0), Vaginal candidiasis (1), Vaginal infection (0), Vaginal mycosis (1), Vaginitis (3), Varicella (2), Vestibular neuronitis (0), Viral rhinitis (1), Viral upper respiratory tract infection (3), Vulvovaginitis (1) |
| Injury, poisoning and procedural complications | Accidental overdose (0), Acute kidney injury (0), Adverse rug reaction (0), Alcohol poisoning (0), Animal bite (2), Anticonvulsant toxicity (1), Back injury (0), Bone contusion (0), Burns first degree (1), Clavicle fracture (0), Cut-finger accident (0), Drug eruption (0), Drug intolerance (1), Drug toxicity (0), Epicondylitis (1), Eye injury (0), Face injury (4), Facial bones fracture (0), Femoral neck fracture (1), Foot fracture (2), Gun shot wound (0), Hand fracture (4), Heat stroke (0), Hip fracture (0), Humerus fracture (0), Hyperthermia (5), Inguinal hernia (0), Injury (2), Injury asphyxiation (0), Jaw fracture (0), Joint dislocation (0), Joint injury (7), Joint luxation (1), Joint sprain (1), Laceration (2), Ligament injury (1), Limb injury (1), Lip injury (2), Lower limb fracture (2), Limb fracture upper (1), Meniscus injury (2), Muscle strain (2), Mouth injury (2), Muscle injury (1), Nicotine abuse (0), Open wound (3), Periorbital hematoma (2), Radius fracture (1), Respiratory fume inhalator disorder (1), Rip fracture (0), Road traffic accident (1), Scratch (1), Skeletal injury (2), Skin injury (1), Spinal cord injury (0), Spinal compression fracture (1), Spinal osteoarthritis (0), Spinal stenosis (0), Subdural hematoma (0), Tobacco abuse (0), Tendon injury (1), Thoracic vertebral fracture (0), Whiplash injury (0), Wrist fracture (0) |
| Investigations | Activated partial thromboplastin time (0), Antinuclear antibodies elevated (0), Biopsy skin (0), Blood alkaline phosphatase abnormal (0), Blood alkaline phosphatase increased (0), Blood folate decreased (0), Blood glucose fluctuation (0), Blood glucose increased (1), Blood iron increased (2), Blood lactate dehydrogenase increased (0), Blood magnesium decreased (1), Blood potassium decreased (0), Blood potassium increased (1), Blood sodium decreased (2), Blood sodium increased (1), Blood testosterone decreased (0), Blood thyroid stimulating hormone decreased (0), Blood triglycerides increased (4), Blood urea increased (0), Cardiac murmur (2), Catheterisation cardiac (1), Creatine elevated (2), Creatine phosphokinase elevated (0), Diagnostic procedure (0), Endoscopy gastrointestinal (0), Endoscopy large bowel (1), Examination (1), Glomerular filtration rate decreased (1), Glucose urine present (2), Haematocrit (0), Haematocrit decreased (2), Haematocrit increased (2), Haemoglobin decreased (0), Haemoglobin increased (2), Haemorrhagic diathesis (1), Hyperhomocysteinaemia (1), Hyperopia (0), Intentional overdose (1), Neurological deterioration (1), Neurological examination abnormal (0), Nuclear magnetic resonance imaging (1), Protein urine present (10), Removal of a medical device (1), Renal function test abnormal (0), Psychotherapy (1), Sleep study (0), Transaminases increased (0), Transient neurological deficit (2), Urine ketone body present (3), Vitamin D decreased (0), Weight improvement (3) |
| Metabolism and nutrition disorders | Adipositas (0), Dehydration (3), Diabetes mellitus (3), Diabetes mellitus inadequate control (2), Diabetes mellitus non-insulin dependent (0), Diabetes mellitus type 1 (0), Eating disorder (0), Electrolyte imbalance (1), Failure to thrive (1), Fluid retention (1), Food craving (1), Glucose tolerance impaired (0), Hypercholesterolemia (7), Hyperglycaemia (0), Hyperlipidaemia (0), Hypertriglyceridemia (1), Hyperuricemia (3), Hypocalcaemia (1), Hypochloraemia (0), Hypoglycaemia (1), Hypokalaemia (3), Hypomagnesaemia (1), Hyponatraemia (1), Hypophosphatemia (0), Hypothyroidism (1), Iron deficiency (1), Obesity (3), Pancreatic steatosis (0), Polydipsia (0) |
| Musculoskeletal and connective tissue disorders | Arthritis (1), Arthropathy (2), Bursitis (2), Buttock pain (1), Chest wall pain (0), Dupuytren’s contracture (1), Excoriation (1), Exostosis (1), Flank pain (0), Goitre (1), Gout (0), Groin pain (1), Hand swelling (1), Hypotonia (2), Intervertebral disc disease (1), Intervertebral disc protrusion (0), Joint effusion (0), Joint range of motion decreased (1), Joint stiffness (1), Joint swelling (3), Limb discomfort (3), Muscular weakness (1), Muscle contracture (1), Muscle cramps (4), Muscle tightness (3), Muscle twitching (4), Musculoskeletal discomfort (1), Musculoskeletal chest pain (0), Neck rigidity (1), Osteitis (1), Osteoarthritis (2), Osteopenia (1), Osteoporosis (1), Pain in jaw (1), Periarthritis (2), Plantar fascilitis (1), Rheumatoid arthritis (1), Rotator cuff syndrome (0), Sensation of heaviness (1), Scoliosis (1), Systemic lupus erythematosus (0), Tendonitis (2), Trigger finger (1) |
| Neoplasms benign, malignant and unspecified | Anaplastic astrocytoma (0), Basal cell carcinoma (1), Brain neoplasm (0), Breast cancer (1), Cyst (0), Eyelid tumor (0), Gammopathy (1), Haemangioma acquired (1), Intraductal papillary mucinous neoplasm (0), Lip neoplasm benign (1), Lipoma (0), Lung neoplasm (0), Lung neoplasm malignant (0), Lymphoma (1), Malignant melanoma (0), Meningioma (0), Metastatic bronchial carcinoma (0), Neoplasm malignant (1), Neoplasm skin (0), Ovarian cancer (1), Pancreatic carcinoma (1), Pancreatic mass (1), Plasma cell myeloma (0), Pulmonary mass (1), Renal cancer (0), Squamous carcinoma (0), Thyroid adenoma (1) |
| Nervous system disorders | Acalculia (1), Acquired epileptic aphasia (0), Ageusia (0), Agraphia (1), Akathisia (1), Apoplexia cerebri (1), Aura (2), Autonomic nervous system imbalance (2), Brain zaps (0), Carotid artery dissection (1), Carpal tunnel syndrome (0), Central nervous system lesion (0), Cerebellar syndrome (2), Cerebral contusion (1), Cerebral cyst (0), Cerebral haemorrhage (0), Cerebral infarction (2), Cerebrovascular accident (2), Cervicobrachial syndrome (0), Clumsiness (0), Cluster headache (1), Complex partial seizures (1), Complex regional pain syndrome (1), Concussion (2), Crying (1), Cubital tunnel syndrome (1), Daily sleepiness (16), Dementia (0), Depressed level of consciousness (1), Dizziness exertional (0), Dizziness postural (4), Dysgraphia (0), Dysphemia (2), Dysphoria (1), Dysstasia (0), Encephalocele (1), Epileptic aura (2), Essential tremor (1), Flapping tremor (1), Forgetfulness (1), Formication (0), Grand mal convulsion (4), Haemorrhage intracranial (0), Head discomfort (2), Hemiparesis (3), Hemiplegia transient (1), High pitched voice (1), Hyperactivity (4), Hyperkinesia (3), Hypersomnia (2), Hyposomnia (1), Hypertonia (1), Hyporeflexia (0), Impatience (1), Increased dyskinesia (3), Increased off condition (4), Increased tremor (5), Intention tremor (0), Interest decreased (1), Ischemic cerebral infarction (0), Loss of consciousness (0), Monoplegia (1), Muscle contractions involuntary (1), Muscle spasticity (0), Motor dysfunction (2), Myoclonus (2), Myoclonic epilepsy (1), Neuralgia (0), Neurological decompensation (1), Nerve compression (0), Neuropathy (0), Neuropathy peripheral (0), Nystagmus (1), Orthostatic intolerance (0), Paralysis (1), Parkinson’s syndrome (0), Parkinsonism (0), Parosmia (1), Partial seizures with secondary generalization (1), Petit mal epilepsy (0), Positive rombergism (0), Post concussion syndrome (0), Post lumbalpunction syndrome (1), Postical headache (1), Postical paralysis (0), Postical state (3), Postural tremor (0), Presyncope (2), Psychomotor retardation (0), Radiculitis (1), Radiculopathy (1), Restless leg syndrome (0), Sciatica (1), Sensory impairment (1), Simple partial seizures (0), Sinus headache (2), Sleepiness (7), Speech disorder (3), Status epilepticus (8), Stroke embolic (0), Synovial cyst (1), Tension headache (2), Thinking abnormal (4), Tic (2), Tongue biting (2), Transient ischemic attack (3), Trigeminal nerve disorder (0), Vocal cord paralysis (0), Word finding (0) |
| Pregnancy, puerperium and perinatal conditions | Abortion induced (0), Pregnancy (1), Pregnancy on oral contraception (0), Premature delivery (0) |
| Psychiatric disorders | Abulia (0), Affective disorder (1), Agitated depression (1), Agoraphobia (0), Alcoholism (0), Amnestic disorder (1), Bizarre sensation (1), Bradyphrenia (1), Claustrophobia (1), Clouded consciousness (0), Communication disorder (1), Conduction disorder (1), Conversion disorder (2), Defiant behaviour (3), Delirium (4), Delusional disorder (2), Depersonalization (0), Depression suicidal (1), Dissociation (1), Distractibility (0), Dyssomnia (1), Excitability (1), Emotional disorder (5), Emotional distress (2), Fear (2), Frustration during cognitive testing (0), Frustration tolerance decreased (1), Generalized anxiety disorder (2), Hallucination auditory (0), Hallucination visual (1), Head banging (0), Hypomanic symptoms (2), Impulsivity (1), Indifference (4), Initial insomnia (11), Middle insomnia (8), Learning disorder (0), Libido increased (0), Listless (0), Negativism (1), Neurosis (0), Nightmare (2), Obsessive-compulsive disorder (0), Paranoia (0), Parasomnia (1), Psychosomatic disease (1), Psychotic disorder due to a general medical condition (0), Self-injurious behaviour (2), Self injurious ideation (0), Sleep decrease (0), Sleep disturbance (3), Sleep quality poor (4), Sleepwalking (0), Social avoidant behaviour (0), Somatization disorder (0), Stress symptoms (3), Suicidal ideation with depression (2), Suicide attempt (2), Suicidality increase (1), Substance abuse relapse (2), Tearfulness (5), Tension (2) |
| Renal and urinary disorders | Bladder disorder (1), Bladder pain (1), Bloody urine (4), Crystalluria (0), Cystitis non infectional (1), Diabetic nephropathy (0), Dysuria (1), Enuresis (1), Genitourinary (1), Glucosuria (2), Haematuria (1), Ketonuria (1), Micturition disorder (0), Micturition urgency (0), Nephrolithiasis (1), Nocturia (1), Pollakiuria (2), Polyuria (2), Proteinuria (2), Renal colic (0), Renal cyst (0), Renal failure (10), Renal failure acute (1), Renal insufficiency (3), Renal pain (1), Urethal meatus stenosis (2), Urinary incontinence (2), Urinary retention (2), Urinary urgency (2), Urine analysis abnormal (1), Uterine cyst (1) |
| Reproductive system and breast disorders | Acquired phimosis (1), Amenorrhoea (5), Benign prostatic hyperplasia (4), Benign soft tissue neoplasm (1), Breast disorder (1), Breast enlargement (0), Breast pain (1), Breast tenderness (1), Ejaculation disorder (0), Genital pain female (1), Gynaecomastia (1), Menopause (0), Menorrhagia (0), Menses delayed (0), Menstrual discomfort (1), Menstruation irregular (1), Metrorrhagia (1), Oedema genital (1), Polycystic ovaries (0), Perineal pain (0), Premenstrual syndrome (0), Priapism (1), Prostatic specific antigen increased (0), Prostatism (0), Prostatitis (0), Scrotal pain (0), Vaginal discharge (1), Vaginal haemorrhage (0), Vulvovaginal discomfort (0) |
| Respiratory, thoracic and mediastinal disorders | Acute respiratory distress syndrome (1), Asthma (5), Bronchitis chronic (0), Bronchospasm (0), Chronic obstructive pulmonary disease (0), Cough increased (11), Dry throat (0), Dysphonia (3), Dyspnoea exertional (3), Haemoptysis (0), Hyperventilation (0), Laryngitis (1), Lung disorder (1), Lung infiltration (0), Maxillary sinusitis (1), Nasal complaints (0), Nasal dryness (0), Nasal valve collapse (1), Paranasal sinus hypersecretion (0), Parosmia (0), Pharyngolaryngeal pain (26), Pneumonia aspiration (1), Pneumonia legionella (0), Postnasal drip (1), Productive cough (0), Pulmonary embolism (1), Pulmonary oedema (0), Pulmonary valve incompetence (1), Respiratory tract congestion (1), Rhinitis allergic (3), Shortness of breath (2), Sinus congestion (5), Sleep apnoea syndrome (0), Snoring (0), Tonsillar disorder (0), Throat tightness (1), Upper respiratory tract congestion (0), Upper respiratory tract inflammation (2), Vocal cord inflammation (1), Wheezing (1) |
| Skin and subcutaneous tissue | Acrodermatitis (1), Actinic keratosis (0), Blister (1), Cellulitis (1), Dandruffs (1), Decubitus ulcer (0), Dermal cyst (0), Dermatitis atopic (0), Dermatitis contact (0), Dyshidrosis (1), Ecchymosis (1), Eczema asteatotic (0), Eczema nummular (1), Erythema (7), Erythema multiforme (1), Exanthem (1), Hair abnormal (0), Heat rush (0), Hyperkeratosis (1), Keratosis pilaris (1), Lichen planus (0), Localized skin reaction (1), Melanocytic naevus (0), Night sweats (3), Onychoclasis (0), Pain of skin (1), Photodermatosis (0), Photosensitivity reaction (1), Pityriasis rosea (1), Pruritus generalized (1), Pruritus genital (0), Psoriasis (2), Purpura (0), Rash generalized (3), Rash maculo-papular (0), Rash papular (1), Rash pruritic (1), Rash psoriaform (0), Rosacea (1), Seborrho (1), Seborrhoeic dermatitis (0), Seborrhoeic keratosis (0), Skin abrasion (2), Skin discolouration (1), Skin exfoliation (0), Skin infection (0), Skin inflammation (0), Skin irritation (1), Skin lesion (2), Skin mass (0), Skin papilloma (0), Skin problems (1), Skin scent abnormal (1), Skin ulcer (1), Sweat gland disease (0), Urticaria (3), Wound hematoma (6), Xeroderma (0) |
| Surgical and medical procedures | Abscess management (0), Ankle arthroplasty (1), Arthroscopic surgery (0), Cardiac pacemaker replacement (0), Carotid endarterectomy (0), Cataract operation (2), Colostomy (1), Coronary arterial stent insertion (0), Ear operation (0), Eye laser surgery (0), Glaucoma surgery (0), Hip surgery (0), Hysterectomy (1), Influenza immunization (0), Meniscus operation (1), Nose operation (0), Nephrectomy (0), Radiotherapy to brain (0), Sinus operation (1), Skin neoplasm excision (1), Spinal fusion surgery (0), Surgery for hallux valgus (1), Toe operation (0), Tooth extraction (0), Tympanoplasty (0), Wart excision (1) |
| Vascular disorders | Arteriosclerosis (0), Blood pressure fluctuation (1), Blood pressure increased (2), Blood pressure systolic increased (0), Blood pressure diastolic increased (1), Deep vein thrombosis (1), Haematemesis (1), Haematoma (3), Hypertensive crisis (4), Hypertensive urgency (1), Hypotension (3), Idiopathic intracranial hypertension (0), Intra-abdominal haematoma (0), Intermittent claudication (1), Orthostatic hypotension (0), Peripheral coldness (0), Phlebitis (0), Pulmonary hypertension (0), Thrombophlebitis (1), Thrombosis (2), Varicose vein (0), Vasovagal episode (0), Venous insufficiency (0) |
| Symptom terms were aggregated according to MedDRA guidance | |

### **Table S8 All reported AEs in less than 5 studies – BRV**

| **System Organ Class** | **Adverse events (frequency)** |
| --- | --- |
| Blood and lymphatic system disorders | Granulocytopenia (1), Lymphadenopathy (1) |
| Cardiac disorders | Angina pectoris (1), Bradycardia (2), Electrocardiogram QT corrected interval prolonged (0), Palpitations (3), Sinus bradycardia (3) |
| Ear and labyrinth disorders | Middle ear inflammation (0), Tinnitus (1) |
| Eye disorders | Diplopia (11), Vision blurred (15) |
| Gastrointestinal disorders | Appendicitis (2), Dyspepsia (7), Gastritis (8), Gastrooesophageal reflux disease (0), Gingival bleeding (1), Stomach pain (3), Toothache (12) |
| General disorders | Chest pain (5), Feeling cold (2), Malaise (6), Oedema peripheral (0) |
| Hepatobiliary disorders | Cholecystic chronic (1), Hepatomegaly (1), Hypertransaminasaemia (1) |
| Immune system disorders | Hypersensitivity (2) |
| Infections and infestations | Asymptomatic bacteriuria (1), Bacteriuria (7), Bronchitis (1), Gastrointestinal infection (1), Gastroenteritis (1), Oral herpes (4), Otitis externa (0), Pharyngitis (0), Pneumonia (6), Respiratory tract infection (2), Rhinitis (1), Influenza (34), Sinusitis (0), Upper respiratory tract infection (43), Vulvovaginal mycotic infection (1) |
| Injury, poisoning and procedural complications | Contusion (14), Excoriation (8), Head injury (10), Joint injury (1), Joint sprain (0), Ligament sprain (7), Limb injury (1), Muscle strain (1), Skin laceration (2) |
| Investigations | Blood triglycerides increased (6), Electrocardiogram ST segment depression (1), GGT increased (12), Hyponatremia (9), Hypophosphateaemia (1), Weight blood cell urine positive (2) |
| Metabolism and nutrition disorders | Anorexia (4), Increased appetite (0) |
| Musculoskeletal and connective tissue disorders | Arthralgia (11), Arthritis (1), Muscle spasm (13), Musculoskeletal chest pain (1), Myalgia (14), Shoulder pain (1) |
| Psychiatric disorders | Agitation (4), Behavioural abnormalities (32), Bradyphrenia (0), Disorientation (0), Euphoria (0), Middle insomnia (2), Mood swings (4), Restlessness (4), Suicide (0), Suicide attempt (0), Suicidal ideation (3) |
| Renal and urinary disorders | Dysuria (3), Enuresis (1), Haematuria (4), Leukocyturia (2), Nephrolithiasis (3), Pollakiuria (0), Renal failure acute (1) |
| Reproductive system and breast disorders | Genital pruritus female (0) |
| Respiratory, thoracic and mediastinal disorders | Cough (22), Dyspnoea (8), Epistaxis (1), Sinus congestion (1), Upper respiratory tract congestion (1) |
| Skin and subcutaneous tissue | Dry skin (0), Ecchymosis (1), Eczema (5), Ingrowing nail (1), Night sweats (0), Pruritus (15), Rash (19), Skin ulcer (0) |
| Vascular disorders | Hematoma (0), Orthostatic hypotension (1), Hot flush (2) |
| Symptom terms were aggregated according to MedDRA guidance | |

### **Fig. S3 Forest plot for headache - LEV vs. PLC**

Headache data from Betts et al. (2000) were not published. Event rates used in the meta-analysis were therefore derived from a pooled adverse event table in the FDA drug approval package, which includes data from Ben-Menachem et al. (2000), Betts et al. (2000), Cereghino et al. (2000), and Shorvon et al. (2000). As headache event rates were reported in the publications by Ben-Menachem et al. (2000), Cereghino et al. (2000), and Shorvon et al. (2000), the corresponding rates for Betts et al. (2000) could be calculated.

### **Fig. S4 Forest plot for somnolence – LEV vs. PLC**

### **Fig. S5 Forest plot for dizziness – LEV vs. PLC**

Dizziness data from Ben-Menachem et al. (2000) were not published. Event rates used in the meta-analysis were therefore derived from a pooled adverse event table in the FDA drug approval package, which includes data from Ben-Menachem et al. (2000), Betts et al. (2000), Cereghino et al. (2000), and Shorvon et al. (2000). As dizziness event rates were reported in the publications by Betts et al. (2000), Cereghino et al. (2000), and Shorvon et al. (2000), the corresponding rates for Ben-Menachem et al. (2000) could be calculated.

### **Fig. S6 Forest plot for fatigue – LEV vs. PLC**

### **Fig. S7 Forest plot for nasopharyngitis – LEV vs. PLC**

### **Fig. S8 Forest plot for nausea – LEV vs. PLC**

Nausea data from N132 (Cereghino et al. (2000)) and N138 (Ben-Menachem et al. (2000)) were not published. Event rates used in the meta-analysis were therefore derived from a pooled adverse event table in the FDA drug approval package, which includes data from Ben-Menachem et al. (2000), Betts et al. (2000), Cereghino et al. (2000), and Shorvon et al. (2000). As nausea event rates were reported in the publications by Betts et al. (2000), and Shorvon et al. (2000), the corresponding pooled rates for N132 (Cereghino et al. (2000)) and N138 (Ben-Menachem et al. (2000)) could be calculated.

Nausea data from Mohs et al. (2023) were not published but were obtained directly from the authors upon request.

### **Fig. S9 Forest plot for diarrhea – LEV vs. PLC**

Diarrhea data from N051 (Shorvon et al. (2000)) and N052 (Betts et al. (2000)) and N138 (Ben-Menachem et al. (2000)) were not published. Event rates used in the meta-analysis were therefore derived from a pooled adverse event table in the FDA drug approval package, which includes data from Ben-Menachem et al. (2000), Betts et al. (2000), Cereghino et al. (2000), and Shorvon et al. (2000). As diarrhea event rates were reported in the publication by Cereghino et al. (2000), the corresponding pooled rates for N051 (Shorvon et al. (2000)) and N052 (Betts et al. (2000) and N138 (Ben-Menachem et al. (2000)) could be calculated.

Diarrhea data from Mohs et al. (2023) were not published but were obtained directly from the authors upon request.

### **Fig. S10 Forest plot for irritability – LEV vs. PLC**

Irritability data from Xiao et al. (20009) were not published by the authors but were obtained from Yie et al. (2018) doi: [10.2147/NDT.S181886](https://doi.org/10.2147/NDT.S181886).

### **Fig. S11 Forest plot for asthenia – LEV vs. PLC**

Asthenia data from Berkovic et al. (2007) were not published but were obtained from Vivli Inc. upon request.

### **Fig. S12 Forest plot for depression – LEV vs. PLC**

Depression data from N052 (Betts et al. (2000)) and N132 (Cereghino et al. (2000)) and N138 (Ben-Menachem et al. (2000)) were not published. Event rates used in the meta-analysis were therefore derived from a pooled adverse event table in the FDA drug approval package, which includes data from Ben-Menachem et al. (2000), Betts et al. (2000), Cereghino et al. (2000), and Shorvon et al. (2000). As depression event rates were reported in the publication by Shorvon et al. (2000), the corresponding pooled rates for N052 (Betts et al. (2000) and N132 (Cereghino et al. (2000)) and N138 (Ben-Menachem et al. (2000)) could be calculated.

Depression data from Berkovic et al. (2007) were not published but were obtained from Vivli Inc. upon request.

Depression data from Glauser et al. (2006) were not published but were obtained from the FDA drug approval package.

### **Fig. S13 Forest plot for insomnia – LEV vs. PLC**

Insomnia data from N051 (Shorvon et al. (2000) and N052 (Betts et al. (2000)) and N132 (Cereghino et al. (2000)) and N138 (Ben-Menachem et al. (2000)) were not published. Event rates used in the meta-analysis were therefore derived from a pooled adverse event table in the FDA drug approval package.

Insomnia data from Berkovic et al. (2007) were not published but were obtained from Vivli Inc. upon request.

Insomnia data from Glauser et al. (2006) and Noachtar et al. (2008) were not published but were obtained from the FDA drug approval package.

### **Fig. S14 Forest plot for vomiting – LEV vs. PLC**

Vomiting data from Berkovic et al. (2007) were not published but were obtained from Vivli Inc. upon request.

Vomiting data from Mohs et al. (2023) were not published but were directly obtained from the authors upon request.

### **Fig. S15 Forest plot for influenza – LEV vs. PLC**

Influenza data from N052 (Betts et al. (2000)) and N138 (Ben-Menachem et al. (2000)) were not published. Event rates used in the meta-analysis were therefore derived from a pooled adverse event table in the FDA drug approval package, which includes data from Ben-Menachem et al. (2000), Betts et al. (2000), Cereghino et al. (2000), and Shorvon et al. (2000). As influenza event rates were reported in the publications by Cereghino et al. (2000) and Shorvon et al. (2000), the corresponding pooled rates for N052 (Betts et al. (2000) and N138 (Ben-Menachem et al. (2000)) could be calculated.

Influenza data from Mohs et al. (2023) were not published but were directly obtained from the authors upon request.

### **Fig. S16 Forest plot for pharyngitis – LEV vs. PLC**

Pharyngitis data from N052 (Betts et al. (2000)) and N132 (Cereghino et al. (2000)) and N138 (Ben-Menachem et al. (2000)) were not published. Event rates used in the meta-analysis were therefore derived from a pooled adverse event table in the FDA drug approval package, which includes data from Ben-Menachem et al. (2000), Betts et al. (2000), Cereghino et al. (2000), and Shorvon et al. (2000). As pharyngitis event rates were reported in the publication by Shorvon et al. (2000), the corresponding pooled rates for N052 (Betts et al. (2000) and N132 (Cereghino et al. (2000)) and N138 (Ben-Menachem et al. (2000)) could be calculated.

Pharyngitis data from Berkovic et al. (2007) were not published but were obtained from Vivli Inc. upon request.

### **Fig. S17 Forest plot for agitation – LEV vs. PLC**

Agitation data from Berkovic et al. (2007) were not published but were obtained from Vivli Inc. upon request.

Agitation data from Mohs et al. (2023) were not published but were directly obtained from the authors upon request.

Agitation data from Noachtar et al. (2008) were not published but were obtained from the FDA drug approval package.

Agitation data from Peltola et al. (20009) were not published by the authors but were obtained from Fang et al. (2013) doi: [10.1016/j.jocn.2013.01.032](https://doi.org/10.1016/j.jocn.2013.01.032).

### **Fig. S18 Forest plot for anxiety – LEV vs. PLC**

Anxiety data from Berkovic et al. (2007) were not published but were obtained from Vivli Inc. upon request.

Anxiety data from Mohs et al. (2023) were not published but were directly obtained from the authors upon request.

Anxiety data from Glauser et al. (2006) and Noachtar et al. (2008) were not published but were obtained from the FDA drug approval package.

### **Fig. S19 Forest plot for rash – LEV vs. PLC**

Rash data from N051 (Shorvon et al. (2000)) and N052 (Betts et al. (2000)) and N132 (Cereghino et al. (2000)) and N138 (Ben-Menachem et al. (2000)) were not published. Event rates used in the meta-analysis were therefore derived from a pooled adverse event table in the FDA drug approval package.

Rash data from Berkovic et al. (2007) were not published but were obtained from Vivli Inc. upon request.

Rash data from Mohs et al. (2023) were not published but were directly obtained from the authors upon request.

### **Fig. S20 Forest plot for accidental injury – LEV vs. PLC**

### **Fig. S21 Forest plot for urinary tract infection – LEV vs. PLC**

Urinary tract infection data from N132 (Cereghino et al. (2000)) and N138 (Ben-Menachem et al. (2000)) were not published. Event rates used in the meta-analysis were therefore derived from a pooled adverse event table in the FDA drug approval package, which includes data from Ben-Menachem et al. (2000), Betts et al. (2000), Cereghino et al. (2000), and Shorvon et al. (2000). As urinary tract infection event rates were reported in the publications by Betts et al. (2000) and Shorvon et al. (2000), the corresponding pooled rates for N132 (Cereghino et al. (2000)) and N138 (Ben-Menachem et al. (2000)) could be calculated.

### **Fig. S22 Forest plot for constipation – LEV vs. PLC**

Constipation data from Berkovic et al. (2007) were not published but were obtained from Vivli Inc. upon request.

### **Fig. S23 Forest plot for abdominal pain – LEV vs. PLC**

Abdominal pain data from N052 (Betts et al. (2000)) and N138 (Ben-Menachem et al. (2000)) were not published. Event rates used in the meta-analysis were therefore derived from a pooled adverse event table in the FDA drug approval package, which includes data from Ben-Menachem et al. (2000), Betts et al. (2000), Cereghino et al. (2000), and Shorvon et al. (2000). As abdominal pain event rates were reported in the publications by Cereghino et al. (2000) and Shorvon et al. (2000), the corresponding pooled rates for N052 (Betts et al. (2000)) and N138 (Ben-Menachem et al. (2000)) could be calculated.

Abdominal pain data from Berkovic et al. (2007) were not published but were obtained from Vivli Inc. upon request.

### **Fig. S24 Forest plot for thrombocytopenia – LEV vs. PLC**

Thrombocytopenia data from Mohs et al. (2023) were not published but were directly obtained from the authors upon request.

### **Fig. S25 Forest plot for headache – BRV vs. PLC**

### **Fig. S26 Forest plot for somnolence – BRV vs. PLC**

### **Fig. S27 Forest plot for dizziness – BRV vs. PLC**

### **Fig. S28 Forest plot for fatigue – BRV vs. PLC**

### **Fig. S29 Forest plot for nasopharyngitis – BRV vs. PLC**

Nasopharyngitis data from Klein et al. (2015) were not published. Event rates used in the meta-analysis were therefore derived from a pooled adverse event table in the FDA drug approval package, which includes data from Biton et al. (2014), Klein et al. (2015) and Ryvlin et al. (2014). As nasopharyngitis event rates were reported in the publications by Biton et al. (2014) and Ryvlin et al. (2014), the corresponding rate for Klein et al. (2015) could be calculated.

### **Fig. S30 Forest plot for nausea – BRV vs. PLC**

Nausea data from Klein et al. (2015) were not published. Event rates used in the meta-analysis were therefore derived from a pooled adverse event table in the FDA drug approval package, which includes data from Biton et al. (2014), Klein et al. (2015) and Ryvlin et al. (2014). As nausea event rates were reported in the publications by Biton et al. (2014) and Ryvlin et al. (2014), the corresponding rate for Klein et al. (2015) could be calculated.

### **Fig. S31 Forest plot for urinary tract infection – BRV vs. PLC**

Urinary tract infection data from Ryvlin et al. (2014) were not published. Event rates used in the meta-analysis were therefore derived from a pooled adverse event table in the FDA drug approval package, which includes data from Biton et al. (2014), Klein et al. (2015) and Ryvlin et al. (2014). As urinary tract infection event rates were reported in the publications by Biton et al. (2014) and Klein et al. (2015), the corresponding rate for Ryvlin et al. (2014) could be calculated.

### **Fig. S32 Forest plot for convulsion – BRV vs. PLC**

Convulsion data from Biton et al. (2014) NCT00464269, Klein et al. (2015) NCT01261325 and Ryvlin et al. (2014) NCT00490035were not published. Event rates used in the meta-analysis were therefore derived from a pooled adverse event table in the FDA drug approval package.

### **Fig. S33 Forest plot for irritability – BRV vs. PLC**

Irritability data from Klein et al. (2015) were not published. Event rates used in the meta-analysis were therefore derived from a pooled adverse event table in the FDA drug approval package, which includes data from Biton et al. (2014), Klein et al. (2015) and Ryvlin et al. (2014). As irritability event rates were reported in the publications by Biton et al. (2014) and Ryvlin et al. (2014), the corresponding rate for Klein et al. (2015) could be calculated.

Irritability data from NCT00175825 were not published. Event rates used in the meta-analysis were therefore derived from a pooled adverse event table in the FDA drug approval package, which includes data from Biton et al. (2014), Klein et al. (2015), Ryvlin et al. (2014), Kwan et al. (2014), Van Paesschen et al, (2013) and NCT00175825. As irritability event rates were reported in the publications by Biton et al. (2014) and Ryvlin et al. (2014) and Kwan et al. (2014) and Van Paesschen et al. (2013) and the rate for Klein et al. (2015) could be defined, the corresponding rate for NCT00175825 could be calculated for the placebo arm and the BRV 50 mg/d arm.

### **Fig. S34 Forest plot for insomnia – BRV vs. PLC**

Insomnia data from NCT0161325 (Klein et al. (2014)) and NCT00490035 (Ryvlin et al. (2014)) were not published. Event rates used in the meta-analysis were therefore derived from a pooled adverse event table in the FDA drug approval package, which includes data from Biton et al. (2014), Klein et al. (2015) and Ryvlin et al. (2014). As insomnia event rates were reported in the publication by Biton et al. (2014, the corresponding pooled rate for NCT0161325 (Klein et al. (2014)) and NCT00490035 (Ryvlin et al. (2014)) could be calculated.

### **Fig. S35 Forest plot for tremor – BRV vs. PLC**

Tremor data from NCT00464269 (Biton et al. (2014)), NCT01261325 (Klein et al. (2015)) and NCT00490035 (Ryvlin et al. (2014)) were not published. Event rates used in the meta-analysis were therefore derived from a pooled adverse event table in the FDA drug approval package.

Tremor data from NCT00504881 (Kwan et al. (2014)), (NCT00175929) Van Paesschen et al. (2013) and NCT00175825 were not published. Event rates used in the meta-analysis were therefore derived from a pooled adverse event table in the FDA drug approval package, which includes data from Biton et al. (2014), Klein et al. (2015), Ryvlin et al. (2014), Kwan et al. (2014), Van Paesschen et al, (2013) and NCT00175825. As pooled tremor event rates could be defined for Biton et al. (2014), Klein et al. (2015) and Ryvlin et al. (2014) the corresponding pooled rate for NCT00504881 (Kwan et al. (2014)), (NCT00175929) Van Paesschen et al. (2013) and NCT00175825 were could be calculated as a pooled rate.

### **Fig. S36 Funnel plot for headache – LEV vs. PLC**

### **Fig. S37 Funnel plot for somnolence – LEV vs. PLC**

### **Fig. S38 Funnel plot for dizziness – LEV vs. PLC**

### **Fig. S39 Funnel plot for nausea – LEV vs. PLC**

### **Fig. S40 Funnel plot for diarrhea - LEV vs. PLC**

### **Fig. S41 Funnel plot for irritability – LEV vs. PLC**

### **Fig. S42 Funnel plot for insomnia – LEV vs. PLC**

### **Fig. S43 Funnel plot for somnolence – BRV vs. PLC**

### **Fig. S44 Funnel plot for dizziness – BRV vs. PLC**

### **Fig. S45 Funnel plot for fatigue – BRV vs. PLC**

### **Table S9 Results of sensitivity analyses**

| **Symptom /**  **Main Analysis** | **Sensitivity analysis:**  **Exclusion of pair with greatest weight** | **Sensitivity analysis: exclusion of pairs with a duration of < 8 weeks** | **Sensitivity analysis: exclusion of pairs with high risk of bias** | **Sensitivity analysis: selective inclusion of pairs with active AE assessment** | **Sensitivity analysis: selective inclusion of pairs with passive AE assessment** | **Sensitivity analysis: exclusion of pairs with any indication of industry sponsorship** | **Sensitivity analysis: exclusion of pairs with pooled data** | **Sensitivity analysis: exclusion of unpublished pairs** |
| --- | --- | --- | --- | --- | --- | --- | --- | --- |
| Headache /  LEV:  20 pairs  OR 0.89 [0.66, 1.21], p=0.46, I²=44%  BRV:  11 pairs  OR 0.82 [0.63, 1.06], p=0.13, I²=0% | LEV:  19 pairs  OR 0.92 [0.66, 1.28], p=0.60, I²=45%  BRV:  10 pairs  OR 0.87 [0.65, 1.17], p=0.36, I²=0% | LEV:  18 pairs  OR 0.89 [0.64, 1.22], p=0.46, I²=44%  BRV:  9 pairs  OR 0.81 [0.62, 1.06], p=0.13, I²=0% | LEV:  17 pairs  OR 1.03 [0.75, 1.40], p=0.88, I²=37%  BRV:  10 pairs  OR 0.82 [0.63, 1.06], p=0.13, I²=0% | LEV:  2 pairs  OR 0.71 [0.23, 2.15], p=0.55, I²=45%  BRV: -* | LEV:  7 pairs  OR 1.04 [0.72, 1.52], p=0.83, I²=13%  BRV:  9 pairs  OR 0.79 [0.61, 1.03], p=0.09, I²=0% | LEV:  13 pairs  OR 0.99 [0.68, 1.43], p=0.95, I²=40%  BRV: - | LEV: -  BRV: - | LEV:  19 pairs  OR 0.92 [0.67, 1.27], p=0.61, I²=45%  BRV: - |
| Somnolence /  LEV:  19 pairs  **OR 1.80 [1.41, 2.30], p<0.00001**, I²=0%  BRV:  10 pairs  **OR 1.86 [1.33, 2.61], p=0.0003**, I²=18% | LEV:  18 pairs  **OR 1.84 [1.42, 2.40], p<0.00001**, I²=0%  BRV:  9 pairs  **OR 1.68 [1.17, 2.41], p=0.005**, I²=5% | LEV:  17 pairs  **OR 1.81 [1.41, 2.33], p<0.00001**, I²=0%  BRV:  9 pairs  **OR 2.09 [1.55, 2.81], p<0.00001**, I²=0% | LEV:  15 pairs  **OR 1.81 [1.38, 2.37], p<0.0001**, I²=3%  BRV: - | LEV:  2 pairs  OR 1.51 [0.67, 3.42], p=0.32, I²=0%  BRV: - | LEV:  6 pairs  **OR 1.79 [1.03, 3.12], p=0.04**, I²=15%  BRV:  9 pairs  **OR 1.88 [1.32, 2.69], p=0.0005**, I²=22% | LEV:  12 pairs  **OR 2.07 [1.51, 2.85], p<0.00001**, I²=0%  BRV: - | LEV: -  BRV: - | LEV: -  BRV: - |
| Dizziness /  LEV:  24 pairs  OR 1.15 [0.84, 1.56], p=0.38, I²=5%  BRV:  11 pairs  **OR 1.75 [1.24, 2.46], p=0.001**, I²=9% | LEV:  23 pairs  OR 1.01 [0.73, 1.39], p=0.96, I²=0%  BRV:  10 pairs  **OR 1.55 [1.08, 2.24], p=0.02**, I²=0% | LEV:  21 pairs  OR 1.18 [0.84, 1.64], p=0.34, I²=11%  BRV:  9 pairs  **OR 2.00 [1.42, 2.81], p<0.00001**, I²=2% | LEV:  20 pairs  OR 1.11 [0.79, 1.58], p=0.54, I²=13%  BRV:  10 pairs  **OR 1.72 [1.21, 2.43], p=0.002**, I²=12% | LEV:  2 pairs  OR 1.23 [0.48, 3.15], p=0.67, I²=0%  BRV: -* | LEV:  9 pairs  OR 1.11 [0.60, 2.08], p=0.74, I²=36%  BRV:  9 pairs  **OR 1.75 [1.22, 2.52], p=0.002**, I²=14% | LEV:  15 pairs  **OR 1.63 [1.08, 2.46], p=0.02**, I²=0%  BRV: - | LEV: -  BRV: - | LEV:  23 pairs  OR 1.16 [0.83, 1.61], p=0.39, I²=9%  BRV: - |
| Fatigue /  LEV:  9 pairs  OR 1.33 [0.81, 2.17], p=0.25, I²=33%  BRV:  10 pairs  **OR 2.14 [1.43, 3.20], p=0.0002,** I²=0% | LEV:  8 pairs  OR 1.02 [0.66, 1.57], p=0.94, I²=0%  BRV:  9 pairs  **OR 1.92 [1.17, 3.14], p=0.010**, I²=0% | LEV: -  BRV:  8 pairs  **OR 2.25 [1.46, 3.47], p=0.0002**, I²=0% | LEV: -  BRV:  9 pairs  **OR 2.11 [1.39, 3.18], p=0.0004**, I²=0% | LEV: -*  BRV: -* | LEV:  4 pairs  OR 0.89 [0.49, 1.61], p=0.70, I²=0%  BRV:  8 pairs  **OR 2.25, [1.45, 3.49], p=0.0003**, I²=0% | LEV:  5 pairs  OR 1.56 [0.72, 3.38], p=0.26, I²=52%  BRV: - | LEV: -  BRV: - | LEV: -  BRV: - |
| Nasopharyngitis /  LEV:  9 pairs  OR 1.26 [0.86, 1.86], p=0.24, I²=8%  BRV:  8 pairs  OR 0.89 [0.59, 1.33], p=0.57, I²=0% | LEV:  8 pairs  OR 1.23 [0.75, 2.04] p=0.41, I²=19%  BRV:  7 pairs  OR 0.90 [0.55, 1.47], p=0.68, I²=0% | LEV:  8 pairs  OR 1.31 [0.91, 1.89], p=0.14, I²=0%  BRV: - | LEV:  8 pairs  OR 1.24 [0.79, 1.93], p=0.35, I²=19%  BRV: - | LEV: -*  BRV: - | LEV:  5 pairs  OR 1.35 [0.86, 2.12], p=0.19, I²=0%  BRV:  7 pairs  OR 0.84 [0.55, 1.29], p=0.43, I²=0% | LEV:  4 pairs  OR 0.66 [0.27, 1.60], p=0.36, I²=31%  BRV: - | LEV: -  BRV: - | LEV: -  BRV:  7 pairs  OR 1.00 [0.63, 1.57], p=0.99, I²=0% |
| Nausea /  LEV:  13 pairs  OR 0.77 [0.52, 1.16], p=0.21, I²=0%  BRV:  8 pairs  OR 1.08 [0.62, 1.89], p=0.79, I²=28% | LEV:  12 pairs  OR 0.74 [0.47, 1.19], p=0.21, I²=0%  BRV:  7 pairs  OR 0.84 [0.51, 1.38], p=0.49, I²=0% | LEV:  12 pairs  OR 0.78 [0.52, 1.17], p=0.23, I²=0%  BRV:  7 pairs  OR 0.79 [0.48, 1.31], p=0.36, I²=0% | LEV:  9 pairs  OR 0.74 [0.45, 1.21], p=0.23, I²=0%  BRV: - | LEV: -*  BRV: - | LEV:  6 pairs  OR 0.78 [0.37, 1.63], p=0.50, I²=0%  BRV:  7 pairs  OR 0.92 [0.51, 1.64], p=0.77), I²=6% | LEV:  8 pairs  OR 0.76 [0.47, 1.23], p=0.26, I²=0%  BRV: - | LEV:  12 pairs  OR 0.83 [0.52, 1.32], p=0.43, I²=0%  BRV: - | LEV:  11 pairs  OR 0.85 [0.53, 1.35], p=0.48, I²=0%  BRV:  7 pairs  OR 0.84 [0.51, 1.38], p=0.49, I²=0% |
| Diarrhea /  LEV:  11 pairs  OR 1.02 [0.70, 1.48], p=0.92, I²=0% | LEV:  10 pairs  OR 1.14 [0.75, 1.73], p=0.54, I²=0% | LEV:  10 pairs  OR 0.99 [0.67, 1.44], p=0.94, I²=0% | LEV:  10 pairs  OR 1.04 [0.68, 1.57], p=0.87, I²=0% | LEV: -* | LEV:  5 pairs  OR 0.95 [0.55, 1.65], p=0.85, I²=0% | LEV:  6 pairs  OR 1.01 [0.60, 1.70], p=0.98, I²=0% | LEV:  10 pairs  OR 1.04 [0.68, 1.57], p=0.87, I²=0% | LEV:  9 pairs  OR 1.04 [0.68, 1.57], p=0.87, I²=0% |
| Irritability /  LEV:  11 pairs  **OR 2.55 [1.41, 4.63], p=0.002,** I²=0%  BRV:  7 pairs  OR 1.60 [0.81, 3.17], p=0.18, I²=0% | LEV:  10 pairs  **OR 4.96 [2.12, 11.61], p=0.0002**, I²=0%  BRV:  6 pairs  OR 1.29 [0.57, 2.92], p=0.54, I²=0% | LEV:  8 pairs  **OR 2.39 [1.26, 4.51], p=0.007**, I²=0%  BRV: - | LEV:  8 pairs  **OR 2.35 [1.26, 4.40], p=0.007**, I²=0%  BRV:  6 pairs  OR 1.74 [0.86, 3.49], p=0.12, I²=0% | LEV:  2 pairs  OR 1.78 [0.61, 5.17], p=0.29, I²=20%  BRV: - | LEV:  6 pairs  **OR 4.50 [1.53, 13.17], p=0.006**, I²=0%  BRV:  6 pairs  OR 1.74 [0.86, 3.49], p=0.12, I²=0% | LEV:  7 pairs  **OR 2.21 [1.12, 4.38], p=0.02**, I²=0%  BRV: - | LEV: -  BRV: - | LEV:  10 pairs  **OR 2.44 [1.33, 4.47], p=0.004,** I²=0%  BRV:  5 pairs  OR 1.32 [0.56, 3.09], p=0.53, I²=0% |
| Asthenia /  LEV:  7 pairs  **OR 1.71 [1.15, 2.54], p=0.008,** I²=0% | LEV:  6 pairs  **OR 1.91 [1.20, 3.05], p=0.007**, I²=0% | LEV:  6 pairs  **OR 1.67 [1.12, 2.49], p=0.01**, I²=0% | LEV:  6 pairs  **OR 1.60 [1.03, 2.49], p=0.04,** I²=0% | LEV: -* | LEV:  2 pairs  OR 1.41 [0.69, 2.90], p=0.35, I²=0% | LEV:  5 pairs  **OR 1.69 [1.10, 2.62], p=0.02**, I²=0% | LEV: - | LEV:  6 pairs  **OR 1.68 [1.13, 2.50],** **p=0.01,** I²=0% |
| Depression /  LEV:  8 pairs  OR 1.62 [0.89, 2.94], p=0.11, I²=0% | LEV:  7 pairs  OR 1.43 [0.68, 3.02], p=0.34, I²=0% | LEV:  7 pairs  OR 1.81 [0.96, 3.44], p=0.07, I²=0% | LEV:  7 pairs  OR 1.43 [0.68, 3.02], p=0.34, I²=0% | LEV: - | LEV:  3 pairs  OR 1.48 [0.40, 5.44], p=0.56, I²=0% | LEV:  5 pairs  OR 1.58 [0.68, 3.67], p=0.29, I²=0% | LEV:  7 pairs  OR 1.43 [0.68, 3.02], p=0.34, I²=0% | LEV:  5 pairs  OR 1.52 [0.64, 3.64], p=0.34, I²=0% |
| Insomnia /  LEV:  11 pairs  OR 1.00 [0.64, 1.56], p=0.99, I²=0%  BRV:  6 pairs  OR 1.19 [0.63, 2.23], p=0.60, I²=0% | LEV:  10 pairs  OR 0.98 [0.57, 1.68], p=0.93; I²=0%  BRV:  5 pairs  OR 0.92 [0.40, 2.08], p=0.83) I²=0% | LEV:  10 pairs  OR 1.00 [0.64, 1.57], p=0.99, I²=0%  BRV: - | LEV:  9 pairs  OR 1.01 [0.58, 1.75], p=0.98, I²=0%  BRV: - | LEV:  2 pairs  OR 1.02 [0.47, 2.24], p=0.95, I²=0%  BRV: - | LEV:  3 pairs  OR 1.22 [0.37, 4.08], p=0.74, I²=0%  BRV:  5 pairs  OR 1.13 [0.58, 2.19] p=0.71, I²=0% | LEV:  7 pairs  OR 0.91 [0.49, 1.68], p=0.76, I²=0%  BRV: - | LEV:  10 pairs  OR 0.98 [0.57, 1.68], p=0.93, I²=0%  BRV:  5 pairs  OR 0.92 [0.40, 2.08], p=0.83) I²=0% | LEV:  7 pairs  OR 1.08 [0.55, 2.13], p=0.82, I²=0%  BRV:  5 pairs  OR 0.92 [0.40, 2.08], p=0.83) I²=0% |
| Vomiting /  LEV:  6 pairs  OR 0.92 [0.52, 1.62], p=0.77, I²=0% | LEV:  5 pairs  OR 0.75 [0.34, 1.67], p=0.48, I²=0% | LEV:  5 pairs  OR 0.92 [0.52, 1.62], p=0.77, I²=0% | LEV:  5 pairs  OR 0.92 [0.52, 1.62], p=0.77, I²=0% | LEV: -* | LEV:  2 pairs  OR 0.35 [0.04, 3.40], p=0.36, I²=N.A. | LEV:  3 pairs  OR 0.93 [0.49, 1.80], p=0.83, I²=0% | LEV: - | LEV:  OR 1.04 [0.57, 1.89], p=0.90, I²=0% |
| Influenza /  LEV:  8 pairs  OR 0.83 [0.52, 1.32] p=0.43, I²=5% | LEV:  7 pairs  OR 0.77 [0.43, 1.37], p=0.37, I²=16% | LEV: - | LEV:  6 pairs  OR 0.74 [0.42, 1.30], p=0.30, I²=10% | LEV: - | LEV:  4 pairs  OR 1.18 [0.68, 2.06], p=0.55, I²=0% | LEV:  3 pairs  OR 0.49 [0.17, 1.45], p=0.20, I²=48% | LEV:  7 pairs  OR 0.84 [0.47, 1.47], p=0.53, I²=18% | LEV:  6 pairs  OR 0.85 [0.46, 1.57], p=0.60, I²=29% |
| Pharyngitis /  LEV:  6 pairs  OR 1.37 [0.82, 2.38], p=0.23, I²=0% | LEV:  5 pairs  OR 1.20 [0.61, 2.39], p=0.60, I²=4% | LEV: - | LEV: - | LEV: - | LEV:  2 pairs  OR 1.96 [0.07, 51.60], p=0.69, I²=56% | LEV:  3 pairs  OR 1.45 [0.72, 2.92], p=0.29, I²=0% | LEV:  5 pairs  OR 1.20 [0.61, 2.39], p=0.60, I²=4% | LEV:  4 pairs  1.27 [0.58, 2.78], p=0.54, I²=16% |
| Agitation /  LEV:  9 pairs  OR 2.02 [0.90, 4.57], p=0.09, I²=0% | LEV:  8 pairs  OR 2.11 [0.74, 6.03], p=0.16, I²=12% | LEV:  8 pairs  OR 2.09 [0.78, 5.62], p=0.14, I²=12% | LEV:  7 pairs  OR 1.83 [0.75, 4.48], p=0.19, I²=2% | LEV: -* | LEV:  8 pairs  OR 2.11 [0.74, 6.03], p=0.16, I²=12% | LEV:  6 pairs  OR 2.40 [0.96, 6.01], p=0.06, I²=0% | LEV: - | LEV:  5 pairs  OR 2.40 [0.96, 6.01], p=0.06, I²=0% |
| Anxiety /  LEV:  9 pairs  OR 0.96 [0.52, 1.77], p=0.89, I²=0% | LEV:  8 pairs  OR 1.64 [0.71, 3.78], p=0.24, I²=0% | LEV:  8 pairs  OR 0.93 [0.50, 1.73], p=0.82, I²=0% | LEV:  8 pairs  OR 0.93 [0.50, 1.73], p=0.82, I²=0% | LEV: -* | LEV:  4 pairs  OR 1.39 [0.43, 4.49], p=0.58, I²=0% | LEV:  6 pairs  OR 0.71 [0.35, 1.43], p=0.34, I²=0% | LEV: - | LEV:  5 pairs  OR 0.80 [0.38, 1.69], p=0.57, I²=0% |
| Rash /  LEV:  7 pairs  OR 0.69 [0.38, 1.27], p=0.23, I²=0% | LEV:  6 pairs  OR 0.66 [0.20, 2.19], p=0.50, I²=0% | LEV:  6 pairs  OR 0.70 [0.38, 1.29], p=0.25, I²=0% | LEV:  6 pairs  OR 0.66 [0.20, 2.19], p=0.50, I²=0% | LEV: -* | LEV:  2 pairs  OR 0.21 [0.01, 4.39], p=0.31, I²=N.A | LEV:  3 pairs  OR 1.26 [0.07, 24.06], p=0.88, I²=42% | LEV:  6 pairs  OR 0.66 [0.20, 2.19], p=0.50, I²=0% | LEV:  4 pairs  OR 0.96 [0.23, 4.00], p=0.96, I²=0% |
| Accidental injury /  LEV:  5 pairs  OR 0.65 [0.36, 1.18], p=0.15, I²=60% | LEV:  4 pairs  OR 0.67, 8030, 1.51], p=0.33, I²=67% | LEV: - | LEV:  3 pairs  OR 0.87 [0.46, 1.65], p=0.67, I²=61% | LEV: - | LEV: -* | LEV:  4 pairs  OR 0.68 [0.34, 1.37], p=0.28, I²=68% | LEV: - | LEV: - |
| Urinary tract infection /  LEV:  7 pairs  OR 0.58 [0.31, 1.11], p=0.10, I²=0%  BRV:  7 pairs  OR 1.05 [0.62, 1.78], p=0.87, I²=0% | LEV:  6 pairs  OR 0.56 [0.26, 1.22], p=0.15, I²=0%  BRV:  6 pairs  OR 1.09 [0.56, 2.13], p=0.80, I²=0% | LEV:  6 pairs  OR 0.56 [0.27, 1.15], p=0.12, I²=0%  BRV: - | LEV:  4 pairs  OR 0.47 [0.21, 1.08, p=0.08, I²=0%  BRV: - | LEV: -  BRV: - | LEV: -  BRV:  6 pairs  OR 0.97 [0.56, 1.69], p=0.93, I²=0% | LEV:  3 pairs  OR 0.35 [0.09, 1.36], p=0.13, I²=37%  BRV: - | LEV:  6 pairs  OR 0.56 [0.26, 1.22], p=0.15, I²=0%  BRV: - | LEV:  6 pairs  OR 0.56 [0.26, 1.22],  P=0.15, I²=0%  BRV:  6 pairs  OR 1.14 [0.66, 1.98], p=0.63, I²=0% |
| Constipation /  LEV:  5 pairs  OR 1.59 [0.54, 4.67], p=0.40, I²=0% | LEV:  4 pairs  OR 1.76 [0.35, 8.73], p=0.49, I²=0% | LEV:  4 pairs  OR 1.76 [0.35, 8.73], p=0.49, I²=0% | LEV: - | LEV: - | LEV:  2 pairs  OR 2.16 [0.19, 24.25], p=0.53, I²=N.A. | LEV:  3 pairs  OR 1.29 [0.35, 4.74], p=0.70, I²=0% | LEV: - | LEV:  4 pairs  OR 1.47 [0.44, 4.91], p=0.53, I²=0% |
| Abdominal pain /  LEV:  5 pairs  OR 0.74 [0.33, 1.64], p=0.45, I²=22% | LEV:  4 pairs  OR 1.15 80.47, 2.82], p=0.76, I²=0% | LEV: - | LEV: - | LEV: - | LEV:  2 pairs  OR 0.62 [0.10, 3.98], p=0.61, I²=40% | LEV:  2 pairs  OR 0.62 [0.19, 2.02], p=0.43, I²=58% | LEV: - | LEV:  4 pairs  OR 0.67 [0.30, 1.50], p=0.33, I²=23% |
| Thrombocytopenia /  LEV:  6 pairs  OR 1.07 [0.60, 1.94], p=0.81, I²=0% | LEV:  5 pairs  OR 1.13 [0.52, 2.43], p=0.76, I²=0% | LEV:  5 pairs  OR 1.14 [0.63, 2.09], p=0.66, I²=0% | LEV:  5 pairs  OR 1.07 [0.60, 1.94], p=0.81, I²=0% | LEV: -* | LEV: -* | LEV:  3 pairs  OR 0.84 [0.30, 2.34], p=0.73, I²=0% | LEV: - | LEV:  5 pairs  OR 1.07 [0.59, 1.96], p=0.82, I²=0% |
| Convulsion /  BRV:  5 pairs  OR 0.65 [0.22, 1.93], p=0.44, I²=60% | BRV:  4 pairs  OR 0.42 [0.11, 1.64], p=0.21, I²=51% | BRV: - | BRV: - | BRV: - | BRV:  4 pairs  OR 1.28 [0.68, 2.41], p=0.44, I²=0% | BRV: - | BRV:  4 pairs  OR 0.42 [0.11, 1.64], p=0.21, I²=51% | BRV:  4 pairs  OR 0.42 [0.11, 1.64], p=0.21, I²=51% |
| Tremor/  BRV:  5 pairs  OR 0.71 [0.35, 1.44], p=0.34, I²=0 % | BRV:  4 pairs  OR 0.47 [0.11, 1.96], p=0.30, I²=0% | BRV:  4 pairs  OR 0.46 [0.13, 1.69], p=0.24, I²=27% | BRV:  4 pairs  OR 0.75 [0.36, 1.54], p=0.43, I²=55% | BRV: -* | BRV:  3 pairs  OR 0.46 [0.09, 2.45], p=0.37, I²=49% | BRV: - | BRV:  3 pairs  OR 0.16 [0.02, 1.12], p=0.06, I²=0% | BRV:  3 pairs  OR 0.16 [0.02, 1.12], p=0.06, I²=0% |

“–*” denotes that no analysis was performed because fewer than two studies were available, precluding a statistically meaningful evaluation. “-” indicates that no sensitivity analysis was conducted, the results are identical with the main analysis.

## **Table S10 Results of comparison – LEV vs. BRV**

| **Symptom** | **LEV** | **BRV** | **Chi-Square** |
| --- | --- | --- | --- |
| Headache | 20 studies  OR 0.89 [0.65, 1.21], p=0.46; I²=44% | 11 studies  OR 0.82 [0.63, 1.06]  p=0.13, I²=1% | Chi²=0.16  p=0.69 |
| Somnolence | 19 studies  OR 1.80 [1.41, 2.30]  p<0.00001, I²=0% | 10 studies  OR 1.78 [1.19, 2.66]  p=0.005, I²=37% | Chi²=0.06  p=0.81 |
| Dizziness | 24 studies  OR 1.15 [0.84, 1.56]  p=0.38, I²=5% | 11 studies  OR 1.77 [1.28, 2.45]  p=0.0006, I²=4% | Chi²=3.56  p=0.06 |
| Fatigue | 9 studies  OR 1.33 [0.81, 2.17]  p=0.25, I²=33% | 10 studies  OR 2.14 [1.43, 3.20]  p=0.0002, I²=0% | Chi²=2.13  p=0.14 |
| Nasopharyngitis | 9 studies  OR 1.26 [0.86, 1.86]  p=0.24, I²=8% | 8 studies  OR 0.89 [0.59, 1.34]  p=0.58, I²=1% | Chi²=1.48  p=0.22 |
| Nausea | 13 studies  OR 0.78 [0.52, 1.18]  p=0.24, I²=0% | 8 studies  OR 1.06 [0.63, 1.81]  p=0.82, I²=0% | Chi²=0.81  p=0.37 |
| Irritability | 11 studies  OR 2.55 [1.41, 4.63] p=0.002, I²=0% | 7 studies  OR 1.60 [0.81, 3.17] p=0.18, I²=4% | Chi²=1.03  p=0.31 |
| Urinary tract infection | 7 studies  OR 0.58 [0.31, 1.11]  p=0.10, I²=0% | 7 studies  OR 1.05 [0.62, 1.78]  p=0.87, I²=0% | Chi²=1.91  p=0.17 |
| Insomnia | 11 studies  OR 1.00 [0.64, 1.56] p=0.99, I²=0% | 6 studies  OR 1.19 [0.63, 2.23] p=0.60, I²=0% | Chi²=0.19  p=0.66 |

## **Table S11 P-values of meta regressions for evaluated AEs for LEV and BRV**

| **LEV** | | **BRV** | |
| --- | --- | --- | --- |
| **Symptom** | **p-value** | **Symptom** | **p-value** |
| Headache | 0.4465 | Headache | 0.3738 |
| Somnolence | 0.4125 | Somnolence | 0.3080 |
| Dizziness | 0.8922 | Dizziness | 0.4629 |
| Fatigue | 0.3930 | Fatigue | 0.6743 |
| Nasopharyngitis | 0.9912 | Nasopharyngitis | 0.5896 |
| Nausea | 0.5522 | Nausea | 0.9155 |
| Diarrhea | 0.5515 | Convulsion | 0.8728 |
| Irritability | N.A. | Irritability | 0.4996 |
| Asthenia | 0.6035 | Urinary tract infection | 0.4599 |
| Depression | 0.2922 | Insomnia | 0.4650 |

## **Fig. S46 Forest plot high dose vs low dose for headache – LEV**

## **Fig. S47 Forest plot high dose vs low dose for somnolence – LEV**

## **Fig. S48 Forest plot high dose vs low dose for dizziness – LEV**

## **Fig. S49 Forest plot high dose vs low dose for headache – BRV**

## **Fig. S50 Forest plot high dose vs low dose for somnolence – BRV**

## **Fig. S51 Forest plot high dose vs low dose for dizziness – BRV**

## **Fig. S52 Forest plot high vs low dose for fatigue – BRV**

## **Fig. S53 Forest plot high dose vs low dose for nasopharyngitis – BRV**

## **Fig. S54 Forest plot high dose vs low dose for nausea – BRV**

## **Fig. S55 Forest plot high dose vs low dose for convulsion – BRV**

## **Fig. S56 Forest plot high dose vs low dose for irritability – BRV**

## **Fig. S57 Forest plot high dose vs low dose for urinary tract infection – BRV**

## **Fig. S58 Forest plot high dose vs low dose for insomnia – BRV**

## **Table S12 Results of subgroup analyses**

| **Symptom** | **Subgroup analysis:**  **grouped by age** | **Subgroup analysis:**  **grouped by indication** | **Subgroup analysis:**  **grouped by titration** | **Subgroup analysis: grouped by background medication** |
| --- | --- | --- | --- | --- |
| Headache | LEV: -*  BRV: - | LEV:  epilepsy (13) vs other (7)  Chi²=0.76, df=1, p=0.38  BRV:  epilepsy (9) vs other (2)  Chi²=0.02, df=1, p=0.88 | LEV:  none (4) vs medium (12) vs slow (2)  Chi²=1.23, df=2, p=0.54  BRV:  none (7) vs any (4)  Chi²=0.93, df=1, p=0.33 | LEV:  none (9) vs other ASM (7)  Chi²=0.54, df=1, p=0.46  BRV: -* |
| Somnolence | LEV:  adults (5) vs paediatric (2)  Chi²=0.00, df=1, p=0.97  BRV: - | LEV:  epilepsy (15) vs other (4)  Chi²=0.43, df=1, p=0.51  BRV: -* | LEV:  none (2) vs medium (12)  Chi²=0.01, df=1, p=0.91  BRV:  none (6) vs any (4)  Chi²=0.69, df=1, p=0.41 | LEV:  none (8) vs other ASM (9)  Chi²=0.07, df=1, p=0.79  BRV: -* |
| Dizziness | LEV:  adults (10) vs paediatric (2)  Chi²=0.23, df=1, p=0.63  BRV: - | LEV:  epilepsy (17) vs other (7)  Chi²=0.03, df=1, p=0.85  BRV:  epilepsy (9) vs other (2)  Chi²=1.33, df=1, p=0.25 | LEV:  none (4) vs medium (12) vs slow (3)  Chi²=0.35, df=2, p=0.84  BRV:  none (6) vs any (4)  Chi²=0.69, df=1, p=0.41 | LEV:  none (9) vs other ASM (9)  Chi²=1.88, df=1, p=0.17  BRV: -* |
| Fatigue | LEV: -*  BRV: - | **LEV:**  **epilepsy (4) vs other (5)**  **Chi²=4.16, df=1, p=0.04**  BRV:  epilepsy (8) vs other (2)  Chi²=0.09, df=1, p=0.77 | LEV: -*  BRV:  none (6) vs any (4)  Chi²=1.28, df=1, p=0.26 | LEV:  none (3) vs other ASM (3)  Chi²=1.52, df=1, p=0.22  BRV: -* |
| Nasopharyngitis | LEV: -*  BRV: - | LEV: -*  BRV: - | LEV: -*  BRV:  none (4) vs any (4)  Chi²=0.77, df=1, p=0.38 | LEV: -*  BRV - |
| Nausea | LEV: -*  BRV: - | LEV:  epilepsy (7) vs other (6)  Chi²=0.00, df=1,p=0.98  BRV: -* | LEV:  none (3) vs medium (5) vs slow (2)  Chi²=2.06, df=2, p=0.36  BRV:  none (4) vs any (4)  Chi²=2.30, df=1, p=0.13 | LEV:  none (6) vs other ASM (2)  Chi²=0.01, df=1, p=0.94  BRV: -* |
| Diarrhea | LEV: -* | LEV:  epilepsy (7) vs other (4)  Chi²=0.32, df=1, p=0.57 | LEV:  none (2) vs medium (4) vs slow (2)  Chi²=1.77, df=2, p=0.41 | LEV:  none (5) vs other ASM (3)  Chi²=0.03, df=1, p=0.85 |
| Irritability | LEV:  adults (9) vs paediatric (2)  Chi²=0.57, df=1, p=0.45  BRV: - | LEV:  epilepsy (5) vs other (6)  Chi²=0.90, df=1, p=0.34  BRV: -* | LEV:  none (2) vs medium (4)  Chi²=1.04, df=1, p=0.31  BRV:  none (5) vs any (2)  Chi²=0.63, df=1, p=0.43 | LEV:  none (5) vs other ASM (3)  Chi²=1.19, df=1, p=0.27  BRV: - |
| Asthenia | LEV: -* | LEV: -* | LEV: -* | LEV:  none (5) vs other ASM (2)  Chi²=1.30, df=1, p=0.25 |
| Depression | LEV: -* | LEV:  epilepsy (6) vs other (2)  Chi²=0.43, df=1, p=0.51 | LEV:  none (2) vs medium (2)  Chi²=0.29, df=1, p=0.59 | LEV:  none (4) vs other ASM (3)  Chi²=0.00, df=1, p=1.00 |
| Insomnia | LEV:  adults (7) vs paediatric (3)  Chi²=0.33, df=1, p=0.57  BRV: - | LEV:  epilepsy (5) vs other (6)  Chi²=0.00, df=1, p=0.97  BRV: - | LEV: -*  BRV:  none (2) vs any (4)  Chi²=2.38, df=1, p=0.12 | LEV:  none (6) vs other ASM (4)  Chi²=0.02, df=1, p=0.90  BRV: - |
| Vomiting | LEV:  adults (3) vs paediatric (2)  Chi²=1.65, df=1, p=0.20 | LEV:  epilepsy (4) vs other (2)  Chi²=1.29, df=1 p=0.26 | LEV: -* | LEV: -* |
| Influenza | LEV: - | LEV:  epilepsy (6) vs other (2)  Chi²=1.06, df=1, p=0.30 | LEV:  none (2) vs medium (3)  Chi²=1.44, df=1, p=0.23 | LEV:  none (3) vs other ASM (3)  Chi²=1.19, df=1, p=0.28 |
| Pharyngitis | LEV:  Adults (4) vs paediatric (2)  Chi²=0.75, df=1, p=0.39 | LEV: - | LEV: -* | LEV: -* |
| Agitation | LEV:  adults (5) vs paediatric (2)  Chi²=0.36, df=1, p=0.55 | LEV:  epilepsy (6) vs other (3)  Chi²=1.53, df=1, p=0.22 | LEV:  none (3) vs medium (3)  Chi²=0.07, df=1, p=0.78 | LEV:  none (3) vs other ASM (5)  Chi²=1.27, df=1, p=0.26 |
| Anxiety | LEV:  adults (6) vs paediatric (2)  Chi²=0.10, df=1, p=0.75 | LEV:  epilepsy (6) vs other (3)  Chi²= 0.98, df=1, p=0.32 | LEV:  none (2) vs medium (2)  Chi²=2.63, df=1, 0.10 | LEV:  none (3) vs other ASM (4)  Chi²=1.39, df=1, p=0.24 |
| Rash | LEV: - | LEV:  epilepsy (4) vs other (3)  Chi²=0.52, df=1, p=0.47 | LEV: -* | LEV:  none (3) vs other ASM (3)  Chi²=0.11 df=1, p=0.74 |
| Accidental injury | LEV: -* | LEV: - | LEV: -* | LEV: -* |
| Urinary tract infection | LEV: -  BRV: - | LEV: -*  BRV: - | LEV: -*  BRV:  none (3) vs any (3)  Chi²=0.12, df=1, p=0.72 | LEV: -*  BRV: - |
| Constipation | LEV: - | LEV:  epilepsy (2) vs other (3)  Chi²=0.00, df=1, p=0.95 | LEV:  none (2) vs slow (2)  Chi²=0.20, df=1, p=0.65 | LEV: -* |
| Abdominal pain | LEV: - | LEV: -* | LEV: -* | LEV:  none (2) vs other ASM (2)  Chi²=0.13, df=1, p=0.72 |
| Tremor | BRV: - | BRV: -* | BRV:  none (2) vs any (2)  Chi²=1.78, df=1, p=0.18 | BRV: - |

“–*” denotes that no analysis was performed because fewer than two studies were available for any group, precluding a statistically meaningful evaluation. “-” indicates that no subgroup analysis was conducted because all included studies belonged to the same category and no studies were available for the comparator subgroup, precluding subgroup comparison.s
